# QSym^2^: A Quantum Symbolic Symmetry Analysis Program for Electronic Structure

**DOI:** 10.1021/acs.jctc.3c01118

**Published:** 2023-12-25

**Authors:** Bang C. Huynh, Meilani Wibowo-Teale, Andrew M. Wibowo-Teale

**Affiliations:** † School of Chemistry, University of Nottingham, Nottingham NG7 2RD, United Kingdom; ‡ Hylleraas Centre for Quantum Molecular Sciences, Department of Chemistry, University of Oslo, P.O. Box 1033 Blindern, N-0315 Oslo, Norway

## Abstract

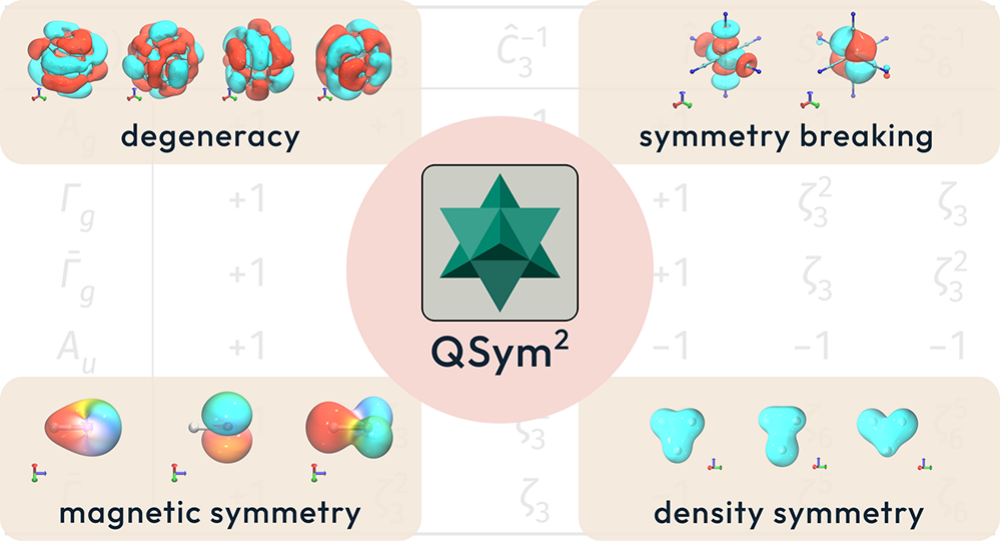

Symmetry provides a powerful machinery to classify, interpret, and understand quantum-mechanical theories and results. However, most contemporary quantum chemistry packages lack the ability to handle degeneracy and symmetry breaking effects, especially in non-Abelian groups, and they are not able to characterize symmetry in the presence of external magnetic or electric fields. In this article, a program written in Rust entitled QSym^2^ that makes use of group and representation theories to provide symmetry analysis for a wide range of quantum-chemical calculations is introduced. With its ability to generate character tables symbolically on-the-fly and by making use of a generic symmetry-orbit-based representation analysis method formulated in this work, QSym^2^ is able to address all of these shortcomings. To illustrate these capabilities of QSym^2^, four sets of case studies are examined in detail in this article: (i) high-symmetry C_84_H_64_, C_60_, and B_9_^–^ to demonstrate the analysis of degenerate molecular orbitals (MOs); (ii) octahedral Fe(CN)_6_^3–^ to demonstrate the analysis of symmetry-broken determinants and MOs; (iii) linear hydrogen fluoride in a magnetic field to demonstrate the analysis of magnetic symmetry; and (iv) equilateral H_3_^+^ to demonstrate the analysis of density symmetries.

## 1 Introduction

Symmetry provides a systematic framework to categorize and classify various mathematical quantities that are of interest to quantum chemists, such as electronic wave functions and densities, via the lenses of group and representation theories. The ability to examine these quantities based on their symmetry enhances one’s arsenal of analysis tools that facilitate the assignment of such quantities calculated from approximate numerical methods to true eigenfunctions of the electronic Hamiltonian of the system. In such studies, having a robust method to unambiguously identify and label the symmetries of the quantities being investigated ensures that their properties can be correctly tracked and assigned to known or expected ground and excited electronic states of the system. This is especially true when the underlying equations that govern such quantities yield multiple solutions with differing degrees of physical relevance, thus making the task of understanding them much more challenging. The simplest and most familiar examples of such equations are the nonlinear self-consistent-field (SCF) Hartree–Fock (HF) and Kohn–Sham (KS) density-functional theory (DFT) equations.^1−6^

In both SCF HF and KS theories, spin–orbitals are one-electron wave functions that form the cornerstones upon which relevant quantities of interest, e.g., single-determinantal wave functions in HF^7^ and electron densities in KS,^8^ are constructed. The spin–orbitals themselves have long been deemed to be of great importance, for they provide chemists with a useful means to interpret the underlying multielectron quantities which are often too complicated to examine directly. In fact, in HF theory, the spin–orbitals that result from the variational optimization of a single-determinantal *ansatz* form the starting point for many families of post-HF correlated methods such as configuration interaction (CI),^9^ coupled cluster (CC),^10^ and complete active space (CAS).^11,12^ On the other hand, in KS theory, there have long been discussions that the KS spin–orbitals are just as useful as their HF counterparts in chemical theories based on molecular-orbital (MO) models [see refs (13 and 14) and also contributions (2.2.4)–(2.2.7) in ref (15)]. In either case, it is imperative that the shape and symmetry properties of spin–orbitals be identified so that they can be used effectively in the qualitative investigations of chemical phenomena^14^ and the quantitative calculations of physical properties such as correlation energies (via post-HF correlated treatments), ionization potentials,^16^ and vertical excitation energies.^17^

However, it is not only the symmetry of spin–orbitals that is important, since spin–orbitals are only one-electron functions and hence do not fully represent electronic states in any multielectron system. In fact, in wave function theories, one often needs to obtain a good understanding of symmetry properties of multielectron wave functions before one can confidently attribute them to actual electronic states of the system, especially when one is interested in more than just the ground state, such as in the computation of electronic spectra.^18,19^ A few studies in which symmetry is used to assist the interpretation of ground- and excited-state correlated wave functions can be found in refs (20−22). In addition, the multiple, generally nonorthogonal, SCF solutions that arise from the HF equations may interact with each other in a CI expansion, if their symmetries are compatible, to give improved multideterminantal wave functions describing certain electronic states with definitive symmetries. Some examples of this include the examinations of low-lying HF solutions in the NO_2_ radical,^23^ in various classes of hydrocarbons,^24^ in octahedral transition-metal complexes,^25^ and in avoided crossings in LiF.^26^ Furthermore, a thorough insight into the symmetry properties of wave functions and densities proves necessary to ensure formal correctness in the fundamental development of DFT and the interpretation of DFT calculation results. This is particularly important in degenerate systems where care must be taken to handle any symmetry breaking in the densities correctly to avoid the well-known symmetry dilemma that often arises in the KS formalism where the KS effective potential has a different symmetry from that of the physical external potential, as discussed in great detail by many authors including Görling,^27,28^ Savin,^29^ and Chowdhury and Perdew.^30^

In addition, since chemistry is hardly ever static, it is often of great interest to follow electronic states as the symmetry of the system is varied. Such a variation can be brought about by various factors such as distortions under vibronic coupling (i.e., Jahn–Teller distortions and related phenomena^31^), mere applications of external magnetic or electric fields,^32−34^ and structural distortions induced by external fields.^35−37^ As the symmetry of the system changes, degeneracies might be lifted and broken symmetry (i.e., when a function and its symmetry partners span multiple irreducible representations of the full symmetry group of the system) might be restored.^25^ A knowledge of wave function and density symmetry allows one to correlate electronic states from low-symmetry configurations to high-symmetry configurations, thus gaining additional insight into their behaviors and properties.

Unfortunately, to the best of our knowledge, despite the importance of symmetry in quantum-chemical theory and computation, there does not yet exist any implementation for a general analysis of symmetry properties of electronic wave functions, densities, and potentials. In fact, many existing general-purpose quantum chemistry packages such as Q-Chem,^38^Orca,^39^PySCF,^40^Dalton,^41^[Open]Molcas,^42^Psi4,^43^CFOUR,^44^ and TURBOMOLE(45) come with features to carry out symmetry analysis to some extent, but most (with Q-Chem and TURBOMOLE being exceptions) opt to work in  or one of its subgroups, all of which are Abelian groups whose irreducible representations are real and one-dimensional, and hence are unable to take into account any spatial degeneracy in wave functions properly. Moreover, none of these packages is able to cope with symmetry breaking, nor are they programmed to examine symmetry properties of quantities other than wave functions, and as far as we are aware, no existing software provides options to analyze symmetry in the presence of external fields.

In this article, a framework for a general symmetry analysis is introduced. This framework is implemented in a Rust^46,47^ program named QSym^2^, which stands for **Q**uantum **Sym**bolic **Sym**metry, and which seeks to address some of the needs for symmetry in electronic-structure theory and computation that are currently not fulfilled by existing quantum chemistry packages. In particular, QSym^2^ is designed to work with *all* possible finite point groups, Abelian or not, for which necessary character tables are automatically and symbolically generated on-the-fly so that degeneracies and symmetry breaking can be represented correctly. In addition, this framework is sufficiently general to be applicable to any linear-space quantities and not just wave functions or densities. Furthermore, QSym^2^ is capable of performing symmetry analysis in the presence of external fields, particularly ensuring that complex irreducible representations, which occur frequently when a magnetic field is present, are handled explicitly. In addition, QSym^2^ is able to provide transformation matrices that enable the generation of symmetry-equivalent partners of any linear-space quantities, as long as they can be expanded in terms of atomic-orbital (AO) basis functions or products thereof. All of this is possible thanks to one governing design principle that QSym^2^ undertakes, which insists that all of its computational elements (e.g., symmetry operations and irreducible representation characters) are treated *symbolically* as much as possible, so that defining properties of groups such as closure and the existence of inverses are respected and utilized to guarantee accuracy and efficiency.

The article is organized as follows. In Section 2, the theoretical foundation for the symmetry analysis framework implemented in QSym^2^ is laid out. In particular, the various aspects of group and representation theories involved in the determination of molecular symmetry groups, the management of symmetry operations, and the *in situ* generation of character tables are explained. This is followed by the formulation of a general method for representation symmetry analysis applicable to any linear space. Then, Section 3 presents several case studies to illustrate the usefulness of symmetry analysis via QSym^2^ in interpreting and understanding electronic-structure calculations. Finally, Section 4 concludes the article with a few remarks on the capabilities and limitations of the symmetry analysis framework implemented in QSym^2^, and also charts possible directions for QSym^2^ to be extended in the future.

## 2 Theory

### 2.1 Symmetry Group Determination

#### 2.1.1 Unitary Symmetry of the Electronic Hamiltonian

For a molecular system with *N*_e_ electrons and *N*_n_ nuclei in a uniform *external* electric field  and magnetic field **B** = **∇** × **A**(**r**), where **A**(**r**) denotes the magnetic vector potential, the electronic Hamiltonian is given by
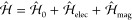
1In atomic units, the first contribution has the form
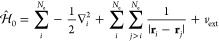
2and is the zero-field Hamiltonian which has an explicit dependence on the multiplicative external potential *v*_ext_ whose form is governed by the geometric arrangement of the nuclei,
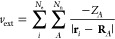
3In Equation 3, **r**_*i*_ denotes the position vector of the *i*^th^ electron and **R**_*A*_ that of the *A*^th^ nucleus. The second contribution,
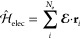
4describes the interaction between the electrons and the external electric field,^48^ and the third contribution,
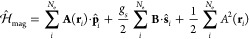
5where **p̂**_*i*_ is the linear momentum operator for the *i*^th^ electron, **ŝ**_*i*_ the spin angular momentum operator for the *i*^th^ electron, and *g*_*s*_ the electron spin *g*-factor, gives the interaction of the electrons with the external magnetic field.^49,50^ The *unitary symmetry group* of the system consists of all unitary transformations *û* that leave  invariant:
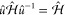
6Clearly,  is the intersection of the unitary symmetry groups of , , and , which we shall denote , , and , respectively. We further restrict the elements in these groups to be *point transformations* acting on the *configuration space* where physical systems such as atoms, molecules, and fields are described.^51^ Then,  is also commonly known as the *point group* of the molecular system.

A robust algorithm to determine the name and elements of  for any molecular system has already been described by Beruski and Vidal.^52^ As shown formally in Appendix A of ref (34), the group  consists of orthogonal transformations in three dimensions [i.e., elements of the group O(3)] that would map the uniform magnetic field **B** onto itself and is commonly known as ,^32,53^ which is an infinite Abelian group with principal axis parallel to **B**. A similar approach can be used to show formally that  consists of three-dimensional orthogonal transformations that would leave the uniform electric field  unchanged and is commonly recognized as ,^32^ which is an infinite, but not Abelian, group with principal axis parallel to . Hence, a naïve procedure to locate all elements of  is to first identify all elements of , and then to filter out only those elements that would leave  and/or **B** invariant. However, this procedure is unnecessarily wasteful as it requires additional efforts to be spent on finding a large number of elements of  that would eventually be discarded, since the presence of external fields almost always leads to a reduction of unitary symmetry. In fact, for highly symmetric molecular systems where  is large, these additional efforts can be nontrivial.

#### 2.1.2 Including External Fields: Method of Fictitious Special Atoms

It is desirable to make use of the algorithm by Beruski and Vidal^52^ as much as possible to locate all elements of  directly without having to go through the intermediary of  in the presence of external fields. To this end, we propose that fictitious special atoms be introduced to represent the external fields such that the combination of the molecule and fictitious atoms has the same unitary symmetry group  as the combination of the molecule and the external fields. Each fictitious special atom is characterized by a pair of parameters (*t*, **R**_*t*_), where *t* encodes its type and **R**_*t*_ denotes its position.

**Figure 1:**
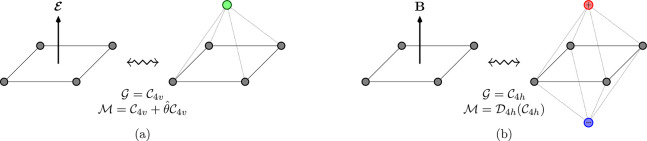
Equivalence between systems in external fields and systems with fictitious special atoms. (a) A single fictitious special atom of type *e* is placed at  to represent a uniform electric field. (b) Two fictitious special atoms, one of type *b*+ and the other of type *b*–, are placed at **R**_com_ ± *k***B** to represent a uniform magnetic field.

A uniform electric field  is represented by one fictitious atom of type *t* = *e* placed at , where **R**_com_ is the position vector of the center of mass of the molecule and *k* a scalar factor chosen to ensure that this fictitious atom does not coincide with any actual atom in the molecule, and that the subsequent unitary symmetry group determination is numerically stable. The vector **R**_*e*_ – **R**_com_ is therefore parallel to , and as  is a polar vector,^54^ it is imposed that fictitious atoms of type *e* transform under all operations in the group O(3) just as any ordinary atom does. It is easily seen that the combination of the molecule and the fictitious atom has the same unitary symmetry group as the molecule in the external  field (Figure 1a).

On the other hand, a uniform magnetic field **B** is represented by two fictitious atoms, one of type *b*+ and the other of type *b*–, placed at **R**_b±_ = **R**_com_ ± *k***B**, where the ± signs in the type names signify the polarities of the fictitious atoms. The vector **R**_*b*+_ – **R**_b–_ is parallel to **B**, and these two fictitious atoms transform under all operations in the group O(3) almost like any ordinary atom, but since **B** is an axial vector,^54^ it is additionally required that the polarities of the fictitious atoms be reversed under improper transformations. This ensures that the combination of the molecule and the fictitious atoms has the same unitary symmetry group as the molecule in the external **B** field (Figure 1b).

**Figure 2:**
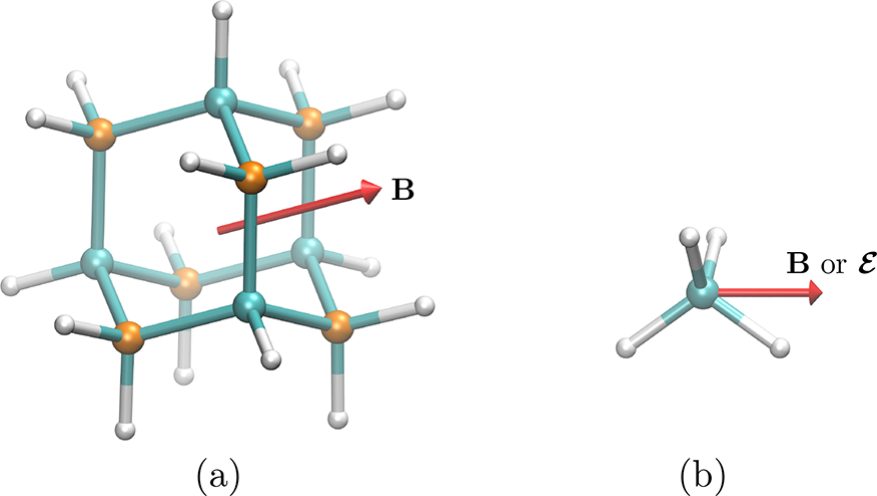
Two special cases involving a uniform external field where the original Beruski–Vidal algorithm^52^ needs to be modified. (a) A tetrahedral adamantane molecule placed in a uniform external magnetic field oriented along one of its *C*_3_ axes. This illustrates a possible scenario in which a polyhedral SEA group (the six carbon atoms highlighted in orange) is arranged in a spherical top fashion (a regular octahedron). (b) A tetrahedral methane molecule placed in a uniform external magnetic or electric field oriented simultaneously parallel to one of the molecular mirror planes and perpendicular to another. This illustrates a possible scenario of the  unitary group arising from the symmetric top rotational symmetry.

With the introduction of fictitious special atoms, external fields are no longer required to be treated separately in unitary symmetry group determination. In fact, fictitious atoms can be incorporated directly into the Beruski–Vidal algorithm,^52^ provided that the following modifications are taken into account:

(i) Fictitious atoms must be included in the calculation of the principal moments of inertia of the system and the subsequent classification into four main rotational symmetry types: spherical top, symmetric top, asymmetric top, and linear. For this purpose, a mass of 100.0u is chosen for the fictitious atoms: there is no physical significance to this value; it simply has been found to ensure numerical stability in all test cases.
(ii) Fictitious atoms must be included in the determination of distance-based symmetrically-equivalent-atom (SEA) groups. This means that *b*+ and *b*– can be in the same SEA group if they both have the same distance signature to all other atoms in the molecule, despite their different polarities.
(iii) The possibility that polyhedral SEAs be arranged in a spherical top must also be taken into account. This additional possibility was not originally considered in ref (52) as it can only arise when a spherical top molecule is placed in an external magnetic field. Figure 2a shows an example where a magnetic field is applied along one of the *C*_3_ axes of tetrahedral adamantane: this molecule-field combined system is now a symmetric top with the unique axis along the field direction, but the six carbon atoms highlighted in orange constitute a group of SEAs that are arranged in a regular octahedron.
(iv) The symmetric top rotational symmetry may also result in the  group. This additional possibility was not considered in ref (52) either as it can only occur when an external field is applied to a spherical top in a manner that eliminates all symmetry elements of the system apart from a single mirror plane. Figure 2b illustrates an example of this when either a magnetic or an electric field is applied to tetrahedral CH_4_ such that it is simultaneously parallel to one of the molecular mirror planes and perpendicular to another.

#### 2.1.3 Magnetic Symmetry of the Electronic Hamiltonian

When antiunitary operations are taken into account, the unitary symmetry group  might no longer be the largest symmetry group of the electronic Hamiltonian . In fact, in many studies involving magnetic phenomena and magnetic materials,^55−59^ it is necessary to consider a supergroup of  that also contains antiunitary symmetry operations that leave  invariant. Such a group is called the *magnetic symmetry group* of the system, and it can easily be seen^56^ that  must admit  as a normal subgroup of index 2, so that we can write
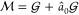
7where *â*_0_ can be any of the antiunitary elements in  but must be fixed once chosen. The left coset  with respect to  contains all antiunitary elements of .

Let us now consider the time-reversal operation θ̂, which is an archetype of antiunitary operations (see Chapter 26 of ref (60) for an in-depth discussion of the time-reversal operation in quantum mechanics). It turns out that, with respect to θ̂, magnetic symmetry groups can be classified into just two kinds.^56,61,62^ The first kind are those that contain θ̂, in which case one can choose *â*_0_ = θ̂ so that
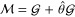
8These are called *magnetic gray groups*. The second kind are those that do not contain θ̂; however, one can always find a unitary operation *û*_0_ not in the group such that the product θ̂*û*_0_ is an antiunitary operation that occurs in the group. This then enables one to write
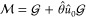
9where *â*_0_ has been chosen to be θ̂*û*_0_. Such groups are called *magnetic black-and-white groups*. It is then clear that, in the absence of an external magnetic field, θ̂ is a symmetry operation of the system. However, this ceases to be the case when an external magnetic field is applied: the magnetic field vector **B** is time-odd^54,56^ and thus gives rise to terms in the electronic Hamiltonian (Equation 5) that do not commute with θ̂ (see Appendix A of ref (34) for a detailed explanation). Therefore, the following general rules can be deduced:

(i) in the absence of an external magnetic field, the system always has a magnetic symmetry group which must be one of the magnetic gray groups;
(ii) in the presence of an external magnetic field, if the system exhibits any antiunitary symmetry, then it has a magnetic symmetry group that must be one of the magnetic black-and-white groups, but if the system exhibits no antiunitary symmetry, then it only has a unitary symmetry group.

For both kinds of magnetic groups, it is often useful to consider a unitary group  that is isomorphic to . In cases where  is identifiable with a subgroup of the full rotation-inversion group in three dimensions O(3) and can thus be given a Schönflies symbol, the magnetic group  can be written as .^59,62^ When this is not possible, however, the antiunitary coset form with respect to the unitary symmetry group  and a representative antiunitary operation *â*_0_ (Equations 7–9) can always be employed to uniquely denote  because it is always possible to assign a Schönflies symbol to , which is guaranteed to be a subgroup of the molecular point group  (cf. Section 2.1.1). Figure 1 depicts two examples of how  is typically denoted.

To determine  and all of its elements given a molecular system in a uniform external field, the Beruski–Vidal algorithm^52^ can once again be exploited with an additional modification that any unitary transformation considered in the algorithm can also be accompanied by the antiunitary action of time reversal. For all ordinary atoms and fictitious atoms of type *e* representing an applied electric field, time reversal has no effects. However, for fictitious atoms of types *b*+ and *b*– representing an applied magnetic field, their polarities must be reversed under time reversal due to the time-odd nature of the magnetic field vector **B**.^54,56^

### 2.2 Abstract Group Construction

#### 2.2.1 Computational Representation of Symmetry Operations

In QSym^2^, symmetry operations located using the method described in the previous section are stored as instances of the SymOp structure. It shall henceforth be written “SymOp(*ĝ*)” to denote an instance of the SymOp structure that represents the actual *ĝ* symmetry operation computationally. In order for this representation to be efficient and to respect discrete-group-theoretic properties, most notably compositability and closure, of the underlying symmetry operations, it is imposed that the SymOp structure fulfill the following traits:

(i) equality comparisons that are equivalence relations—reflexivity, transitivity, and symmetry must be satisfied for the “=” relation between SymOp instances, which must take into account the 2π-periodicity of spatial rotations;
(ii) hashability—each SymOp(*ĝ*) instance must be able to produce an integer hash value hash[SymOp(*ĝ*)] that allows itself to be looked up from a hash table with an average constant time *O*(1), and that must be compatible with equality comparisons: SymOp(*ĝ*_1_) = SymOp(*ĝ*_2_) ⇒ hash[SymOp(*ĝ*_1_)] = hash[SymOp(*ĝ*_2_)];
(iii) compositability—SymOp(*ĝ*_1_) * SymOp(*ĝ*_2_) = SymOp(*ĝ*_1_*ĝ*_2_) where “*” denotes the composition operation between two SymOp instances.

The design of the SymOp structure in QSym^2^ is detailed in Section S1 of the Supporting Information to illustrate how the above traits are satisfied.

#### 2.2.2 Unitary Conjugacy Class Structure

Prior to the generation of the character table of the symmetry group, its conjugacy class structure must first be determined. The conjugacy class structure of a group, in turn, depends on how the conjugacy equivalence relation between group elements is defined. In this article, only the familiar *unitary conjugacy equivalence relation* is considered:
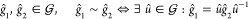
10which holds when all elements in the group are represented as *mathematical* unitary operators on linear spaces, even if some of them are actually *physical* antiunitary operators. A different conjugacy equivalence relation called *magnetic conjugacy equivalence relation* holds if some of the elements in the group are represented on linear spaces as mathematical antiunitary operators,^63^ which leads to a different conjugacy class structure.^63,64^ Although magnetic conjugacy classes have also been implemented in QSym^2^, their uses in magnetic symmetry via corepresentation theory^60^ will be examined in a future study.

The classification of elements of finite molecular symmetry groups in QSym^2^ is carried out via the *Cayley table***C** of the group:
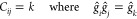
11which encodes the group’s multiplicative structure in a two-dimensional array of integers. The compositions *ĝ*_*i*_*ĝ*_*j*_ are effected computationally through the corresponding compositions SymOp(*ĝ*_*i*_) * SymOp(*ĝ*_*j*_) of the SymOp structure. Once the Cayley table **C** has been computed and stored, any operations that call for the multiplicative structure of the group, such as the determination of the conjugacy class structure or the construction of the group’s character table (Section S2 of the Supporting Information), only need to make cheap queries to **C** without having to repeatedly recalculate group element compositions.

### 2.3 Generation of Character Tables of Irreducible Representations

Once an abstract group structure has been obtained for the underlying symmetry group, its character table then must be computed to allow for subsequent symmetry analysis. This can indeed be performed on-the-fly in QSym^2^. Algorithms for the automatic generation of symbolic character tables^65−67^ are well-known and have been implemented before, most notably in GAP.^68^ However, no such implementation exists for molecular symmetry applications in quantum chemistry. These algorithms are thus reimplemented in QSym^2^ with additional functionalities to ensure that the generated character tables respect conventions that are familiar to most chemists, e.g., the labeling of irreducible representations using Mulliken symbols.^69^ The details of these algorithms are recapitulated in Section S2 of the Supporting Information.

The ability to generate character tables automatically and symbolically enables QSym^2^ to completely circumvent the need for hard-coded character tables, which would limit the number and types of groups available for symmetry analysis. In particular, as demonstrated in Section 3.1, QSym^2^ is able to handle degeneracy in non-Abelian symmetry groups which are encountered in highly symmetric molecular structures such as disk-like boron clusters (),^70,71^ hydro-clusters of group-14 elements in quantum dots (),^72,73^ and buckminsterfullerenes (). QSym^2^ is also capable of tackling complex-valued representations that frequently arise in the presence of an external magnetic field, e.g., eight out of the 12 one-dimensional irreducible representations in , which is the unitary symmetry group of benzene in the presence of a uniform perpendicular magnetic field, are complex.

In all cases, there is no requirement for the molecule and/or external fields to be in any predefined standard orientation for the character-table generation algorithm to work. In fact, as long as the symmetry operations of the system can be computationally represented and composited as described in Section 2.2.1 and Section S1 of the Supporting Information, the group structure can be abstracted away from these concrete representations of symmetry operations to allow for the character table to be computed entirely algebraically without recourse to any other knowledge exterior to the group structure. Only when *labels* of computed irreducible representations are to be deduced is information about molecular structures and symmetry operation orientations required in order to satisfy Mulliken’s conventions. This ensures that molecules and external fields can be placed in whatever orientation is most sensible or convenient for chemical computation, while still being able to benefit from the symmetry analysis offered by QSym^2^.

### 2.4 Representation Analysis

An initial formulation of the method for representation analysis has been discussed by one of the authors in a previous article (Appendices B and C of ref (25)). However, this formulation only focuses on wave functions in Hilbert spaces and therefore leaves out other quantum-chemical quantities that are not wave functions but that still have symmetry properties. Examples of such quantities include electron densities, vibrational coordinates, and magnetically induced ring currents. In this section, a more general formulation of this method will be presented in which *all* linear-space quantities are covered. It will also be pointed out how the computational availability of group multiplicative structures via Cayley tables leads to a reduction in the representation analysis time complexity from  to  by taking advantage of group closure.

#### 2.4.1 Formulation of Linear-Space Representation Analysis

##### 2.4.1.1 Characters and Representation Matrices

Let *V* be a linear space and **w** an element in *V* whose symmetry under a prevailing group  is to be computationally determined. To this end, the linear subspace *W* ⊆*V* that is spanned by the orbit
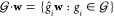
12where *ĝ*_*i*_ denotes the action of *g*_*i*_ on *V* is first determined. The symmetry of **w** in  is then given by the decomposition of *W* into known irreducible representation spaces on *V* of the group .

Technically, *ĝ*_*i*_ and *g*_*i*_ are two very different quantities: the former is an operator acting on *V* and thus a member of GL(*V*) (i.e., the group of all general linear operators acting on *V*), whereas the latter is a member of an abstract group . However, this distinction is unnecessarily pedantic for the purpose of this article and will therefore be ignored: we will use the hatted forms almost exclusively, refer to them as members of the group , and make no attempt to distinguish operators that represent actions of the same abstract element *g*_*i*_ but on different linear spaces.

To characterize *W*, we seek its character function χ^*W*^ whose value for each element *ĝ* in the group is given by
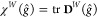
where **D**^*W*^(*ĝ*) is the representation matrix of *ĝ* in some finite basis chosen for *W*. Let  be such a basis. The elements of **D**^*W*^(*ĝ*) satisfy the set of equations
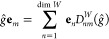
13one for each element **e**_*m*_ in the basis.

##### 2.4.1.2 Representation Matrix Determination

Equation 13 now needs to be solved in a suitably chosen basis to determine the diagonal *D*_*nn*_^*W*^(*ĝ*) elements so that the character value χ^*W*^(*ĝ*) can be computed. In principle, these equations can be viewed as a set of simultaneous equations that can be solved algebraically to give the required matrix elements. However, such an approach would be tedious, and it is much more common for equations of this type to be solved using a projection operator, which requires the existence of an inner product.

Thus far, no reference has been made to any inner products on *V*, because inner products are not required in the definition of representation symmetry. In fact, symmetry is a linear-space property rather than an inner-product-space property. This realization has an important implication: we are at liberty to *define* any inner product that is the most convenient to compute for a given linear space *V* in order to construct a projection operator solely for the purpose of inverting Equation 13; the value of the character χ^*W*^(*ĝ*) must be independent of this choice of inner product, even if *V* itself does not possess an intrinsic inner product.

Let us now endow *V* with an inner product ⟨·|·⟩ with which the overlap matrix **S** between elements in the orbit  is defined:
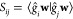
14It would then be ideal to use the orbit  as a basis  for *W* with which Equation 13 can be solved to give the character values. However, the elements in  are not necessarily linearly independent, and the matrix **S** is thus not necessarily of full rank. In this case, a tall rectangular matrix **X** can be constructed:
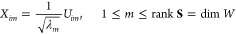
15where λ_*m*_ is a nonzero eigenvalue of **S** and *U*_*im*_ the *i*^th^ component of the corresponding eigenvector. The matrix **X** allows a linearly independent basis for *W* to be defined:
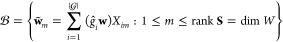
16such that the overlap matrix in this basis,
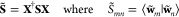
is of full rank and Equation 13 becomes
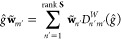
17where primed subscripts have been used for later convenience. If **S** is already of full rank, then we simply set , and so **S̃** = **S**. In either case, the square matrix **S̃** is invertible, with which a nonorthogonal projection operator *P̂*_*m*_ can be constructed:^74^
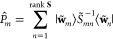
18where *S̃*_*mn*_^–1^ = (**S̃**^–1^)_*mn*_. This projection operator satisfies
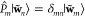
19Applying *P̂*_*m*_ to both sides of Equation 17 and making use of Equation 19 gives
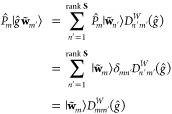
Multiplying both sides by ⟨**w̃**_*m*_| and using the definition of *P̂*_*m*_ in Equation 18 then yields
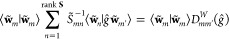
or equivalently, by canceling out the ⟨**w̃**_*m*_|**w̃**_*m*_⟩ term on both sides,
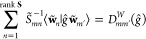
By reintroducing the original terms in the orbit  using Equation 16, we obtain
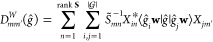

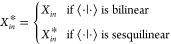
The above result can be conveniently written in a matrix form:
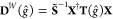
20where
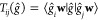
21which gives a closed-form expression for the representation matrix **D**^*W*^(*ĝ*) to be computed from elements in the orbit .

##### 2.4.1.3 Optimization by Group Closure

It is clear from Equation 20 that the computation speed of **D**^*W*^(*ĝ*) is limited by the computation speed of the orbit overlap matrix **S** and the matrices **T**(*ĝ*), for all . Naïvely, explicit constructions of **S** based on Equation 14 and of **T**(*ĝ*) based on Equation 21 would incur time complexities of  and , respectively. However, closure of  under composition allows all matrix elements of **S** and **T**(*ĝ*) to be identifiable with only  unique values:
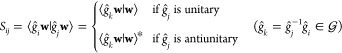
22and
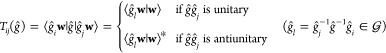
23

In both cases, as long as the overlaps between the elements in the orbit  and the orbit origin **w** have been evaluated, which costs  time, all matrix elements of **S** and **T**(*ĝ*) can be deduced without any further involvement of the expensive overlap computation. However, this optimization is only possible if one can make the identifications *ĝ*_*k*_ = *ĝ*_*j*_^–1^*ĝ*_*i*_ (Equation 22) and *ĝ*_*l*_ = *ĝ*_*j*_^–1^*ĝ*^–1^*ĝ*_*i*_ (Equation 23), which require knowledge of the multiplicative structure of the group . The implementation of SymOp in QSym^2^ that enables the construction of the Cayley table (Equation 11) achieves exactly this and allows QSym^2^ to perform extremely efficient representation analysis of linear-space quantities.

#### 2.4.2 Examples of Linear-Space Representation Analysis

##### 2.4.2.1 Single-Determinantal Wave Functions and Spin–Orbitals

Let us consider an *N*_e_-electron single-determinantal wave function:
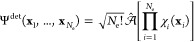
24In the above expression,
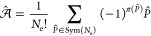
is the antisymmetrizer with *P̂* an element of , the symmetric group of degree *N*_e_, and π(*P̂*) the parity of *P̂*. The antisymmetrizer acts on the electronic spin-spatial coordinates **x**_*i*_ in terms of which the spin–orbitals χ_*i*_ are written. In these cases, the linear space *V* (cf. Section 2.4.1.1) is chosen to be an *N*_e_-particle Hilbert space denoted . It should be noted that spin–orbitals χ_*i*_ are special cases of Ψ^det^ with *N*_e_ = 1. Being a Hilbert space,  comes equipped with the familiar inner product
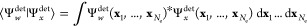
25which can be leveraged to compute the orbit overlap matrix **S** (Equation 14) for Ψ^det^ under the action of the prevailing group . In particular, for single determinants, the inner product in Equation 25 has a particularly simple form:^75^
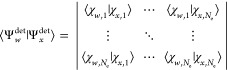
26where χ_*w*,*i*_ denotes the *i*^th^ occupied spin–orbital of the Ψ_*w*_^det^ determinant. Each spin–orbital can be expanded in terms of the AO basis functions according to
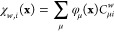
where φ_μ_(**x**) is an AO spin-spatial basis function and μ a composite spin-spatial index. The required spin–orbital overlaps can then be written as
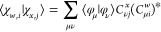
where the two-center overlap integrals
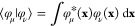
27can be easily obtained from many available integral packages for Gaussian AO basis functions (e.g., Libint(76) and Libcint(77)) or London AO basis functions (e.g., QUEST,^78^London,^79^BAGEL,^80,81^ and ChronusQ(82)). QSym^2^ also implements its own generic *n*-center overlap integral routine based on the recursive algorithm by Honda et al.^83^ that is capable of handling both Gaussian and London AO basis functions.^84^

The calculation of overlaps between single determinants (Equation 26) is available in QSym^2^, thus enabling the symmetry analysis of single-determinantal wave functions and spin–orbitals. Analogous overlap calculations for multideterminantal wave functions are in principle possible, but currently not yet implemented in QSym^2^.

##### 2.4.2.2 Electron Densities

Let us consider next an *N*_e_-electron density for an *N*_e_-electron wave function Ψ(**x**_1_, ..., **x**_*N*_e__):^8,85^
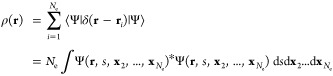
where the composite spin-spatial coordinate **x**_1_ has been relabeled and separated into a spin coordinate *s* and a spatial coordinate **r** in the integrand. In an AO basis, ρ(**r**) can be expanded as
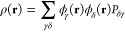
28where ϕ_γ_(**r**) and ϕ_δ_(**r**) are spatial AO basis functions, γ and δ spatial indices, and *P*_*δγ*_ elements of the corresponding density matrix **P** in this basis.

The containing linear space for ρ(**r**) is well-known to be the Banach space .^85^ Being a Banach space,  does not have an intrinsic inner product, but, as explained in Section 2.4.1, it is possible to endow  with an inner product for the purpose of representation analysis. The simplest such inner product can be defined as follows:
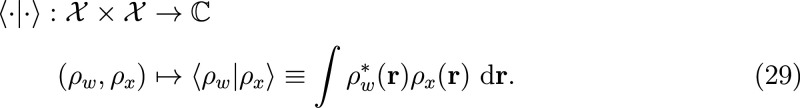
29It is straightforward to show that the above definition for ⟨·|·⟩ satisfies all required properties of an inner product:

- conjugate symmetry:
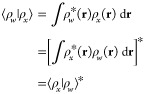
- linearity in the second argument:
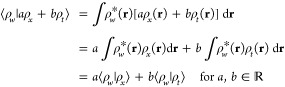
- positive-definiteness:
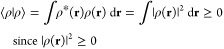
where ⟨ρ|ρ⟩ = 0 if and only if ρ(**r**) = 0 identically; otherwise there would exist regions in  where |ρ(**r**)|^2^ < 0, which is not possible.

Using the basis-expanded form of the electron density in Equation 28 in the inner product definition in Equation 29 gives
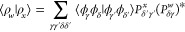
where
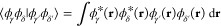
are four-center overlap integrals computable using the generic *n*-center overlap integral routine as described earlier.

The calculation of overlaps between electron densities (Equation 29) is available in QSym^2^, thus enabling the symmetry analysis of electron densities obtained from a wide range of electronic-structure methods from single- and multideterminantal wave functions to DFT.

## 3 Results and Discussion

In this section, several case studies showcasing the capabilities and utility of QSym^2^ are presented. Each case study is based on a distinct computational chemical problem whose results can be better understood by a detailed analysis of symmetry provided by QSym^2^.

### 3.1 Degeneracy in Non-Abelian Groups

**Figure 3:**
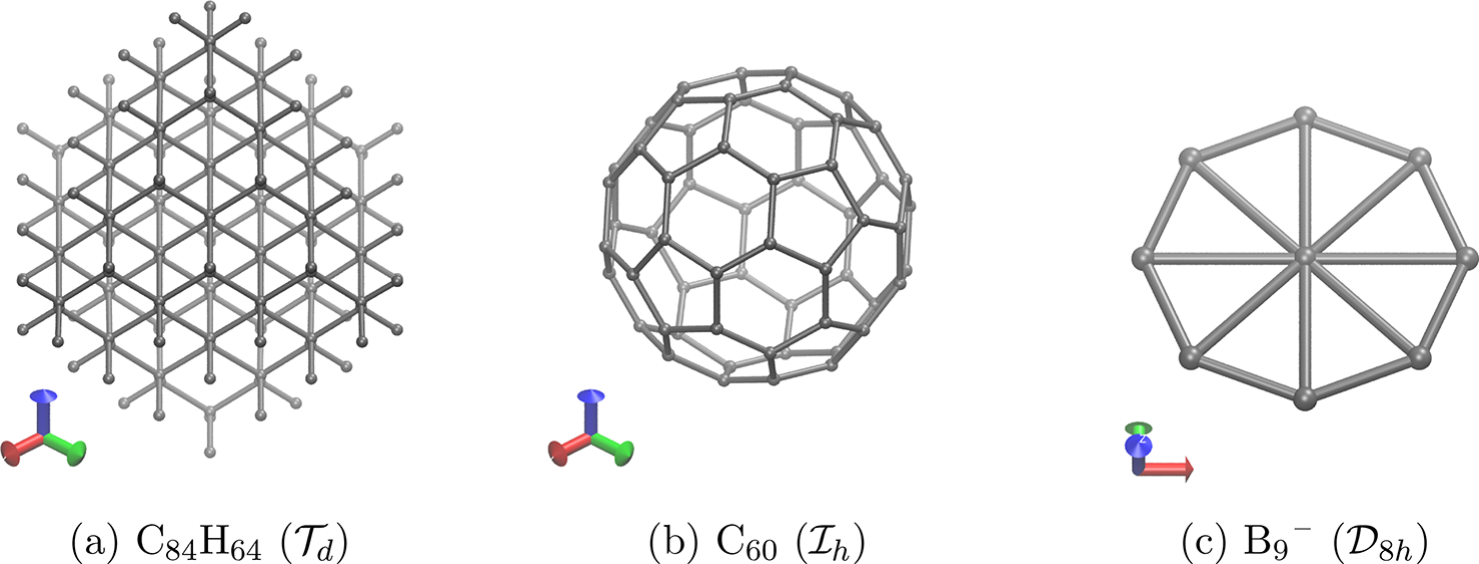
High-symmetry molecules whose ground-state KS MOs exhibit degeneracy.

The first set of case studies consists of three molecules of various sizes and symmetries: a tetrahedral C_84_H_64_ quantum dot (Figure 3a), an icosahedral C_60_ (Figure 3b), and an octagonal B_9_^–^ (Figure 3c). The non-Abelian symmetry of these three molecules allows for degeneracy to occur in the SCF MOs that arise from either a HF or a KS-DFT description of the ground state of the system. By examining the symmetry of such degenerate MOs in the ground SCF solutions of these three molecules, we sought to demonstrate the capability of QSym^2^ to determine degenerate irreducible representation labels accurately, irrespective of the size of the molecules or the complexity of the MOs.

#### 3.1.1 Computational Details

For each molecule, a ground-state KS-DFT calculation using an appropriate exchange-correlation functional and basis set was performed in Q-Chem 6.1.0. In all calculations, suitable symmetry thresholds were chosen to ensure that Q-Chem produced symmetry assignments for the computed MOs in the highest possible group. Afterward, all geometry information, basis set information, and MO coefficients from these calculations were passed to QSym^2^ where the unitary symmetry group  of the system was deduced, following which the representations of  spanned by the MOs were identified and analyzed according to the formulation given in Section 2.4. The MO symmetry assignments from QSym^2^ were then compared with those from Q-Chem.

Q-Chem was chosen as the benchmarking program for these case studies because of its ability to assign degenerate symmetry labels in certain non-Abelian groups. As stated in Section 1, most other quantum-chemistry programs are able to perform symmetry analysis only in Abelian groups and are thus not suitable for this purpose.

#### 3.1.2 Degenerate Symmetry Benchmarks

We begin with tetrahedral C_84_H_64_, which proves to be a straightforward case. Using the geometry reported by Karttunen et al.,^72^ a ground-state unrestricted CAM-B3LYP/6-31+G* calculation was performed and a set of KS MOs were obtained. Table 1 shows the symmetry assignments that have been produced by both Q-Chem and QSym^2^ for the frontier MOs. These particular MOs have been chosen because they have been identified in ref (73) to be responsible for the most intense transition in the electronic absorption spectrum of this molecule, and are therefore the most interesting to examine from a symmetry perspective. For this system, Q-Chem is able to identify its symmetry group as , as is QSym^2^. Both programs are also able to agree on their symmetry assignments of the frontier MOs, degenerate or not, thus confirming that the representation analysis formulation implemented in QSym^2^ (Section 2.4) is valid.

**Table 1:**
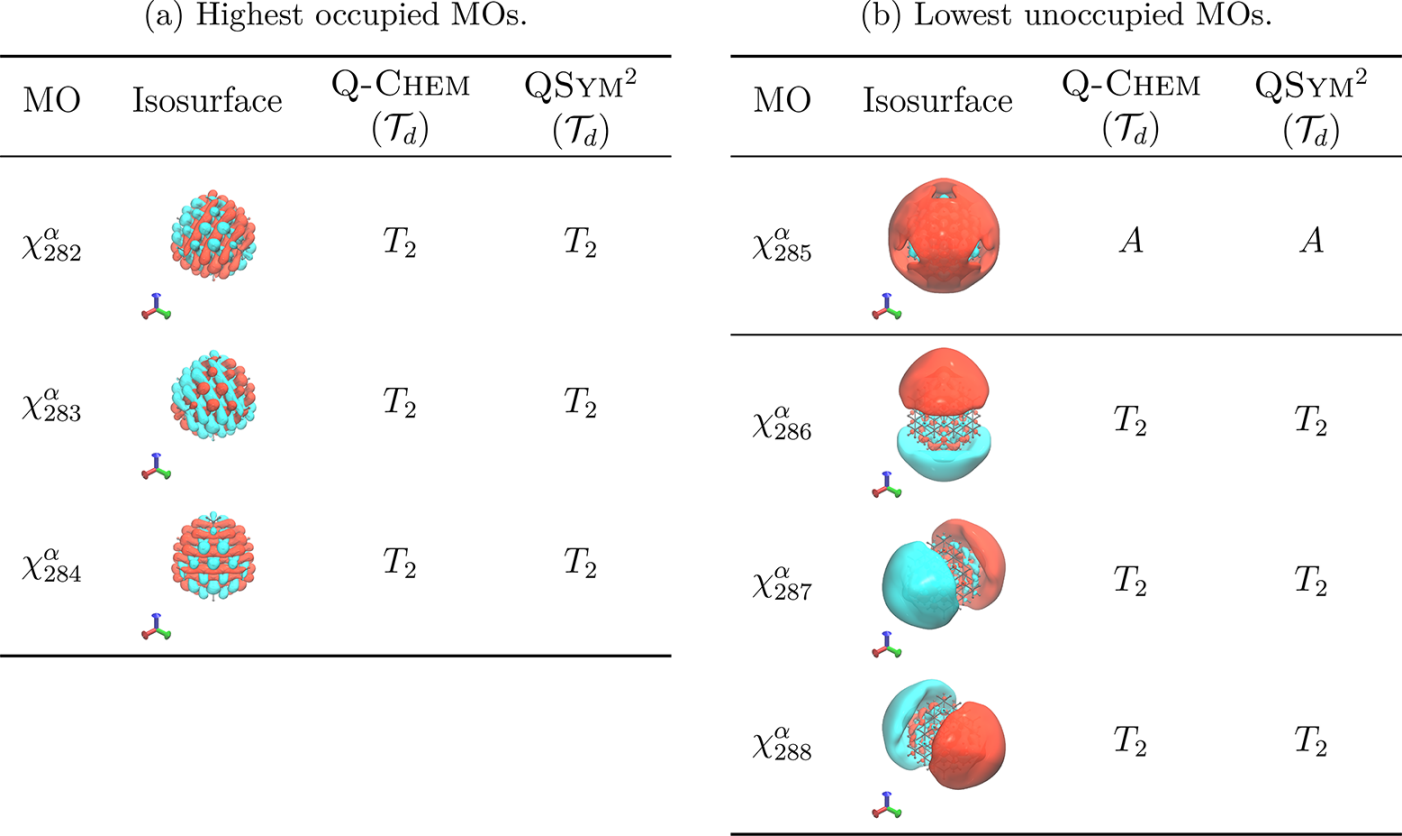
Comparison of Symmetry Assignments of Frontier Canonical MOs in C_84_H_64_ Calculated at the CAM-B3LYP/6-31+G* Level of Theory Using the Geometry Reported by Karttunen et al.^72^^,^^a^

a For each MO χ(**r**), the isosurface is plotted at |χ(**r**)| = 0.008.

We consider next icosahedral C_60_, which has a higher symmetry than tetrahedral C_84_H_64_, and for which an unrestricted CAM-B3LYP/6-31+G* calculation was also carried out to yield a set of KS MOs. It turns out that, even though Q-Chem is able to identify the symmetry group of this system as  when the symmetry tolerance value is set at 1 × 10^–4^, it seeks recourse to  (a subgroup of ) to perform symmetry analysis, but then fails to produce any symmetry assignments for almost all MOs except those that are nondegenerate. Tightening the symmetry tolerance value to 1 × 10^–5^ forces Q-Chem to identify the symmetry group as  instead, but this then allows for a successful assignment of symmetry labels to MOs, albeit only under . On the other hand, QSym^2^ is able to both identify the symmetry group correctly as  and classify the symmetry of MOs using the irreducible representations of . Table 2 shows these symmetry assignments for the three highest degenerate sets of occupied MOs.

**Table 2:**
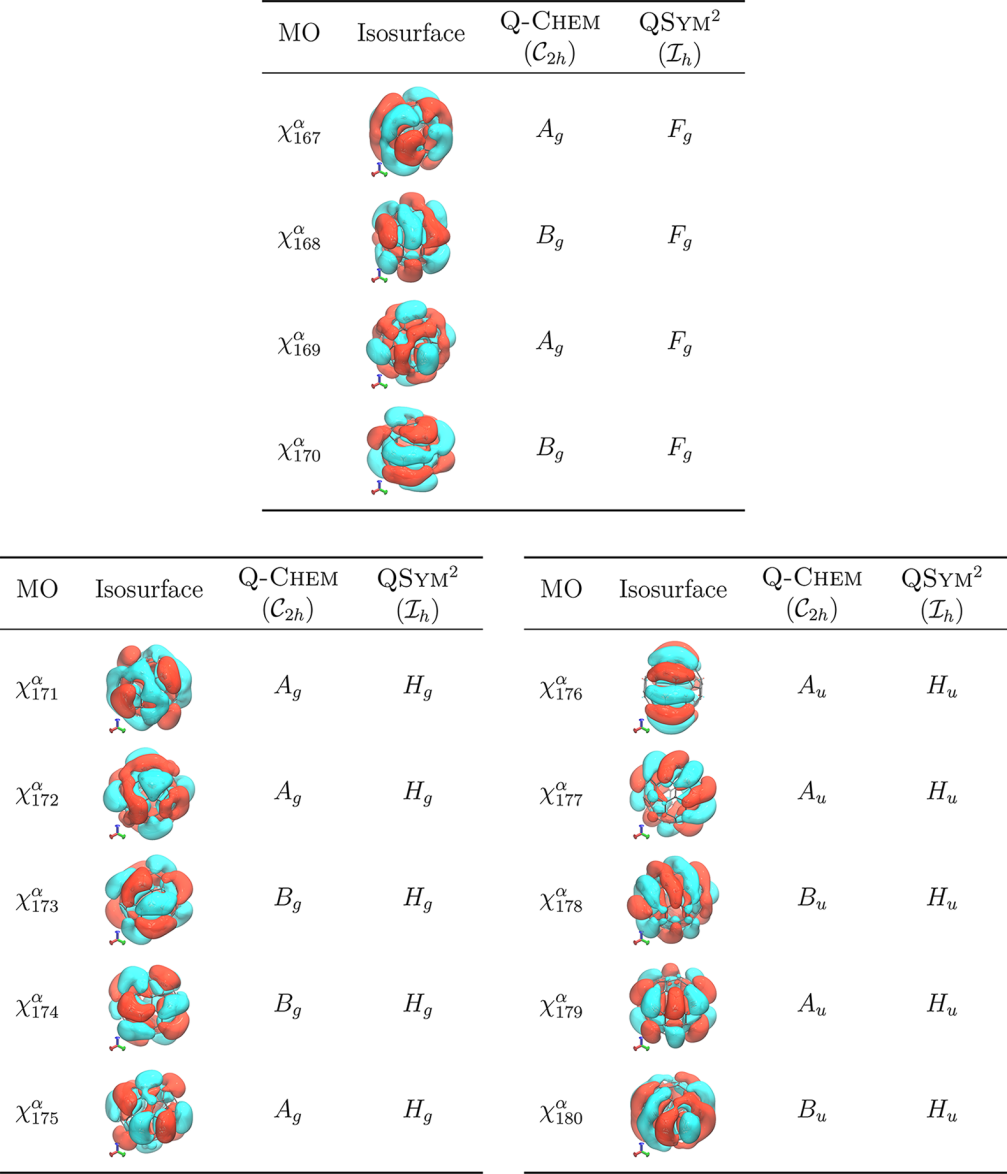
Comparison of Symmetry Assignments of Frontier Occupied Canonical MOs in C_60_ Calculated at the CAM-B3LYP/6-31+G* Level of Theory Using an -Symmetrized Geometry^a^

a For each MO χ(**r**), the isosurface is plotted at |χ(**r**)| = 0.008. The four- and five-dimensional irreducible representation labels follow the convention specified in ref (86).

**Figure 4:**
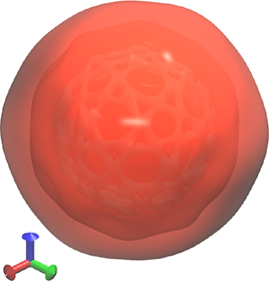
Isosurface plot of the virtual canonical MO χ_195_^α^ at |χ_195_^α^(**r**)| = 0.009 in C_60_ calculated at the CAM-B3LYP/6-31+G* level of theory using an -symmetrized geometry (see Table 2). This MO has  and  symmetry.

An inspection of Table 2 makes clear the necessity of correct symmetry classifications of these MOs in the full group of the underlying molecule. Consider for instance the transition dipole moment integral ⟨χ_176_^α^|**μ̂**|χ_195_^α^⟩, where **μ̂** is the dipole moment operator and χ_195_^α^ the first totally symmetric virtual α-MO in the calculation shown in Table 2 (Figure 4). In , the dipole moment operator transforms as *A*_*u*_ ⊕ 2*B*_*u*_, and so the integrand transforms as *A*_*u*_ ⊗ (*A*_*u*_ ⊕ 2*B*_*u*_) ⊗*A*_*g*_ ⊃ *A*_*g*_, indicating that the integral ⟨χ_176_^α^|**μ̂**|χ_195_^α^⟩ contains up to one independent nonvanishing component. This would thus lead one to the incorrect expectation that the transition χ_195_^α^ ← χ_176_^α^ is optically allowed. However, in , the dipole moment operator transforms as *T*_1*u*_, and the above integral is part of a degenerate set ⟨χ_*i*_^α^|**μ̂**|χ_195_^α^⟩, *i* = 176, ..., 180 whose integrands transform as *H*_*u*_ ⊗ *T*_1*u*_ ⊗ *A*_*g*_ which does not contain *A*_*g*_. The transitions χ_195_^α^ ← χ_*i*_^α^, *i* = 176, ..., 180 are therefore all optically forbidden in . This example shows that such MO symmetry considerations, when done accurately in the full symmetry group of the system, can improve the efficiency of algorithms that make use of symmetry to screen molecular integral evaluations^87−90^ or sharpen the interpretation of spectroscopic results.

Finally, we consider an octagonal boron disc, B_9_^–^, which has  symmetry and should, in principle, be simpler than the  symmetry of C_60_. Unfortunately, no matter the symmetry tolerance value, Q-Chem is only able to determine the symmetry group of this anion as  and classify the MOs calculated at the B3LYP/def2-TZVP level of theory using the irreducible representations of this group. QSym^2^, on the other hand, is capable of identifying the symmetry group as either  or , depending on the choice of the distance threshold (Section S1.4 of the Supporting Information), and then assigning MO symmetry labels using the irreducible representations of the corresponding groups. These are summarized in Table 3 for the valence canonical MOs of B_9_^–^ as reported by Đorđević et al.^71^ It can be seen that, in , the symmetry assignments from both Q-Chem and QSym^2^ are in agreement, while those in  computable only by QSym^2^ provide a full symmetry classification of the MOs that is consistent with their nodal structures.

**Table 3:**
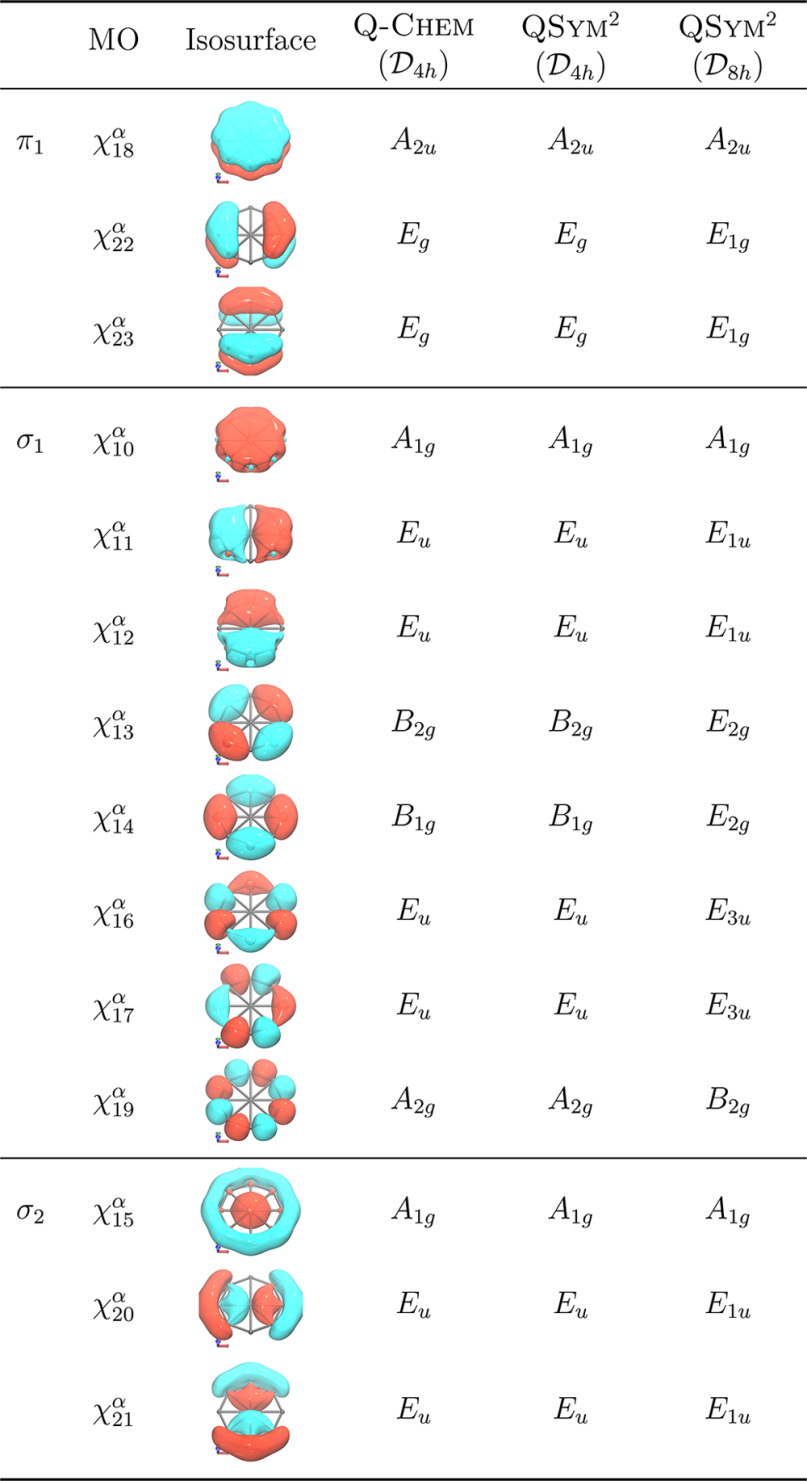
Comparison of Symmetry Assignments of Valence Canonical MOs in B_9_^–^ Calculated at the B3LYP/def2-TZVP Level of Theory Using the Geometry Optimized at the Same Level by Đorđević et al.^71^^,^^a^

a For each MO χ(**r**), the isosurface is plotted at |χ(**r**)| = 0.04. In QSym^2^, the distance thresholds yielding  and  are 10^–5^ and 10^–4^, respectively (see Section S1.4 of the Supporting Information for an explanation of this threshold).

### 3.2 Symmetry Breaking in SCF Solutions and Orbitals

The next case study seeks to demonstrate the ability of QSym^2^ to detect and quantify symmetry breaking in SCF solutions and orbitals. For this purpose, the octahedral complex Fe(CN)_6_^3–^ has been chosen. Having an unpaired electron due to the low-spin *d*^5^ configuration on the Fe^3+^ metal center surrounded by an octahedral ligand field, this complex is expected to give rise to multiple low-lying SCF UHF solutions, some of which break spatial symmetry.^25^

#### 3.2.1 Computational Details

In all calculations, the structure of Fe(CN)_6_^3–^ was held fixed at an  geometry with Fe–C = 2.0256023 Å and C–N = 1.1570746 Å. Multiple SCF solutions at the UHF/def2-TZVP level of theory were found for this geometry in Q-Chem 6.1.0 using metadynamics^91^ combined with the Direct Inversion in the Iterative Subspace (DIIS) algorithm.^92^ SCF convergence was set at a DIIS error value of 1 × 10^–10^ as implemented in Q-Chem. For each converged solution, its geometry information, basis set information, and Pipek–Mezey-localized^93,94^ MO coefficients were read in by QSym^2^ from which symmetry assignments for the individual MOs, as well as for the overall wave function, were determined. Unfortunately, no benchmarking symmetry assignments were available because no existing programs were able to handle symmetry-broken quantities.

For each quantity that is symmetry-analyzed in QSym^2^, a threshold λ_**S**_^thresh^ must be chosen to determine which eigenvalues of the orbit overlap matrix **S** (Equation 14) are nonzero so that the transformation matrix **X** can be constructed (Equation 15). Whether a particular choice of threshold is reasonable depends on the gap between the eigenvalue of **S** that is immediately above the threshold, λ_**S**_^>^, and the eigenvalue of **S** that is immediately below the threshold, λ_**S**_^<^. In all cases for Fe(CN)_6_^3–^, the threshold was chosen such that log_10_ λ_**S**_^>^ – log_10_ λ_**S**_^<^ ≳ 4.

#### 3.2.2 Symmetry Breaking in Fe(CN)_6_^3–^

Table 4a presents the energies of four lowest-lying *M*_*S*_ = +1/2 UHF solutions of Fe(CN)_6_^3–^ alongside their symmetry assignments from QSym^2^ determined at the linear independence threshold λ_**S**_^thresh^ = 1 × 10^–7^. All four solutions are found to be symmetry-broken (i.e., they each span a reducible representation space of ), and interestingly, the lowest two solutions, A and B, do not contain the *T*_2*g*_ term naïvely expected of the ground state for a *d*^5^ configuration in an octahedral strong-field environment,^95^ whereas the other two solutions, C and D, do. In addition, the A and B solutions have different inversion symmetry (*ungerade*) compared to that of the C and D solutions (*gerade*). This strongly suggests that there is a qualitative difference between these two pairs of solutions.

**Table 4:**
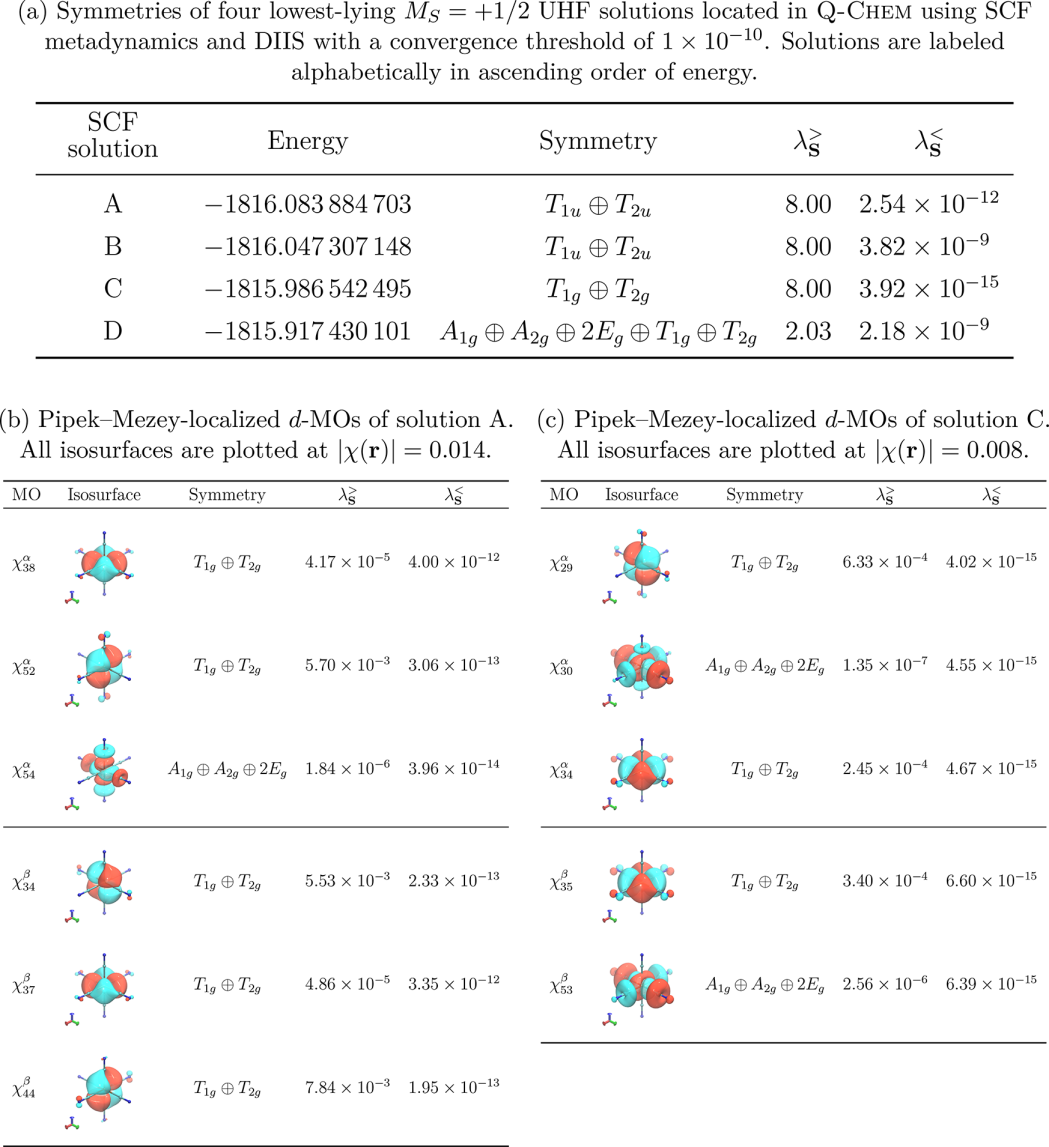
Symmetry-Broken SCF Solutions of Fe(CN)_6_^3–^ Calculated at the UHF/def2-TZVP Level of Theory Using an  Geometry with Fe–C = 2.0256023 Å and C–N = 1.1570746 Å^a^

a Each solution has *M*_*S*_ = +1/2. All symmetries were determined using QSym^2^ with a linear independence cut-off λ_**S**_^thresh^ = 1 × 10^–7^. See Section 3.2.1 in the main text for the description of λ_**S**_^>^ and λ_**S**_^<^.

For illustrative purposes, we focus only on the lower-energy solution in each of the two pairs, namely, the A and C solutions. To acquire a crude understanding of the origin of this qualitative difference, we turn to the Pipek–Mezey-localized MOs^93,94^ obtainable from the canonical MOs of these two solutions, mainly because localized MOs have been known to provide a useful link between detailed quantum-chemical calculations and classical chemical concepts such as nonbonding orbitals, lone pairs, and multiple bonds with which most chemists have gained great familiarity and intuition.^96,97^ In the particular case of Fe(CN)_6_^3–^, localized orbitals help quantify the number of *d*-electrons on the iron center and allow for a discussion on the nature of the UHF solutions in terms of the metal *d*^*n*^-electronic-configuration and oxidation-state descriptors that are common in coordination chemistry.

In Table 4b and Table 4c, the Pipek–Mezey-localized *d*-MOs for the A and C solutions are listed, respectively. Each of these MOs has a *d*-shell Mulliken population of at least 0.980 and can therefore be regarded as being predominantly contributed by a *d*-electron on the iron center. Clearly, the iron center in solution A admits a *d*^6^ configuration, whereas that in solution C admits a *d*^5^ configuration. This is also confirmed by a LOBA oxidation-state analysis formulated by Thom et al.:^97^ the iron center in solution A has an oxidation state of +2, whereas that in solution C has an oxidation state of +3. Noting that all *d*-orbitals on the iron center must have *gerade* inversion symmetry, and also that all *p*-orbitals on the iron center have been confirmed to be paired, we conclude that the *ungerade* inversion symmetry in solution A must arise from an unmatched *ungerade* ligand orbital between the two spin spaces. The seemingly innocent *ungerade* inversion symmetry found in solution A turns out to be a manifestation of a ligand-to-metal charge transfer process.

The fact that the four UHF solutions A–D are symmetry-broken means that none of them is able to provide a physical description of the ground state of the system.^98^ However, it has been demonstrated elsewhere^25^ that post-HF methods such as NOCI can yield multideterminantal wave functions that conserve symmetry and thus provide more appropriate approximations of the ground state. For this to be viable, either a basis  spanning a complete representation space *W* (Equation 16) or a full symmetry-equivalent orbit spanning the same space (Equation 12) must be provided as the basis for NOCI—both of which can be generated by QSym^2^.

**Figure 5:**
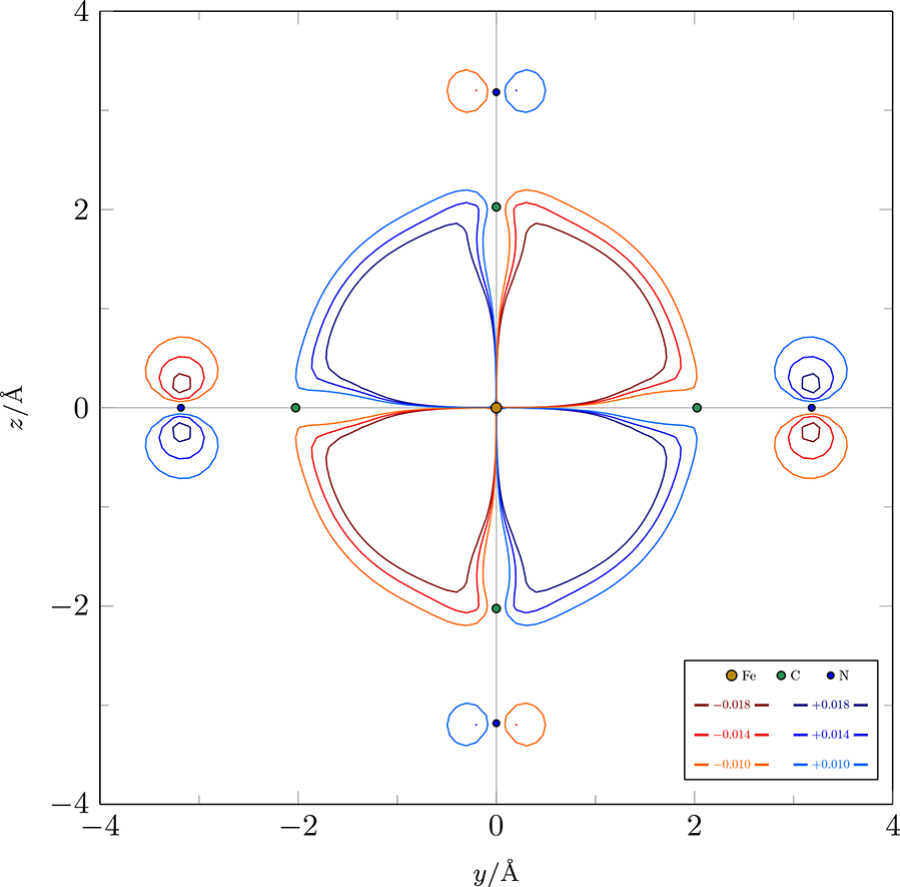
Contours of the Pipek–Mezey-localized MO χ_34_^β^ of solution A in the *yz*-plane. The inequivalence between the interactions of the (CN)^−^ π orbitals in the *y*- and *z*-directions with the Fe-based *d*_*yz*_ orbital accounts for the *T*_1*g*_ ⊕*T*_2*g*_ symmetry breaking of this MO.

We conclude this case study with a remark that as expected, the symmetry breaking of the overall determinants shown in Table 4 can be traced back to the symmetry breaking of the constituting orbitals. This is in fact demonstrated by the symmetry assignments for the Pipek–Mezey-localized *d*-MOs of the A and C solutions in Table 4b,c, respectively. It should be noted, however, that symmetry breaking effects can sometimes be subtle and difficult to discern from a mere visual inspection of the isosurface plots. A detailed analysis based on the formulation in Section 2.4 should therefore be preferred to obtain reliable symmetry information. For instance, consider the MO χ_34_^β^ of solution A whose isosurface at 0.014 is shown in Table 4b. At first glance, this orbital appears just like a typical *d*_*yz*_ orbital (with some distortions due to interactions with the ligands) and should just have *T*_2*g*_ symmetry. However, a close inspection of this isosurface, with the aid of the contour plot in the *yz*-plane shown in Figure 5, reveals that the interactions with the ligands on the *y*-axis are not equivalent to those on the *z*-axis, thus causing the relation *Ĉ*_4_^*x*^χ_34_^β^ = −χ_34_^β^ to fail to hold. In other words, χ_34_^β^ and *Ĉ*_4_^*x*^χ_34_^β^ are linearly independent, which gives rise to the *T*_1*g*_ ⊕*T*_2*g*_ symmetry breaking as observed. The large gap between the boundary orbit overlap eigenvalues λ_**S**_^>^ and λ_**S**_^<^ (ca. 10 orders of magnitude) indicates that this symmetry breaking is in fact not just a numerical artifact of the analysis but rather an intrinsic feature of this MO.

### 3.3 Symmetry in External Fields

Thus far, we have demonstrated the symmetry analysis capability of QSym^2^ for real orbitals and determinants in the absence of any external fields. In this next case study, we illustrate how QSym^2^ can be used to understand quantum-chemical behaviors when external fields are introduced. In particular, we shall show that, for a hydrogen fluoride molecule in a uniform magnetic field, a knowledge of the symmetries of the complex-valued MOs helps rationalize the reversal of the electric dipole moment along the internuclear axis observed by Irons et al.^34^ at strong fields perpendicular to the molecule but not, curiously, at parallel fields.

#### 3.3.1 Computational Details

We followed Irons et al.^34^ and performed current-DFT calculations with the cTPSS functional in the uncontracted aug-cc-pVQZ basis set^99,100^ employing the resolution-of-the-identity approximation with the AutoAux auxiliary basis^101^ in QUEST.^78^ The obtained KS MOs were then passed to QSym^2^ for symmetry analysis in the appropriate unitary symmetry group  of the molecule-plus-field system (cf. Section 2.1.3). In all cases, the gauge origin of the magnetic field and the center of mass of the molecule were placed at the origin of the Cartesian coordinate system so that the gauge origin would always be left invariant by the applications of all symmetry operations during the orbit generation (Equation 12).^102^ For the parallel and perpendicular field orientations, complex MO isosurfaces were also plotted in VMD(103) using the method described by Al-Saadon et al.^104^

**Figure 6:**
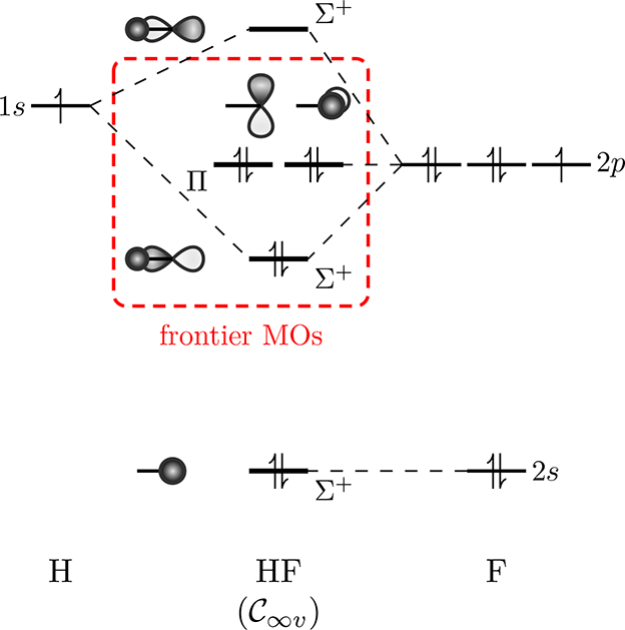
Simplistic depiction of the MOs in hydrogen fluoride at zero field.

In the cases where  is an infinite group (i.e.,  at zero field or  at parallel-field orientations), a finite integer order *n* ≥ 2 is chosen for the infinite-order rotation axis *C*_∞_ so that  is restricted to a suitable finite subgroup  (i.e.,  or , respectively) in which the representation analysis of Section 2.4 is carried out by QSym^2^. The actual representations in  can then be deduced by the representations in  produced by QSym^2^ according to the following subduction rules:
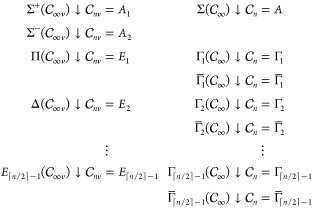
where Γ_*k*_ and Γ̅_*k*_ in  and  are complex-conjugate one-dimensional irreducible representations with character functions
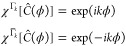
For each choice of integer *n* ≥ 2, irreducible representations up to and including  in  and  and  in  remain irreducible in the respective subgroups  and . These irreducible representations in  can therefore be deduced unambiguously from those in . In the current case study of hydrogen fluoride, it is known from basic MO theory (Figure 6) that MOs of up to Π symmetry at zero field are occupied in the ground state, and so we require *n* ≥ 3 so that  and  have enough irreducible representations to describe , , and  symmetries unequivocally. In fact, for good measure, we chose *n* = 8 in all infinite-group symmetry analyses for hydrogen fluoride in QSym^2^.

#### 3.3.2 MO Description of Electric Dipole Reversal in a Magnetic Field

**Figure 7:**
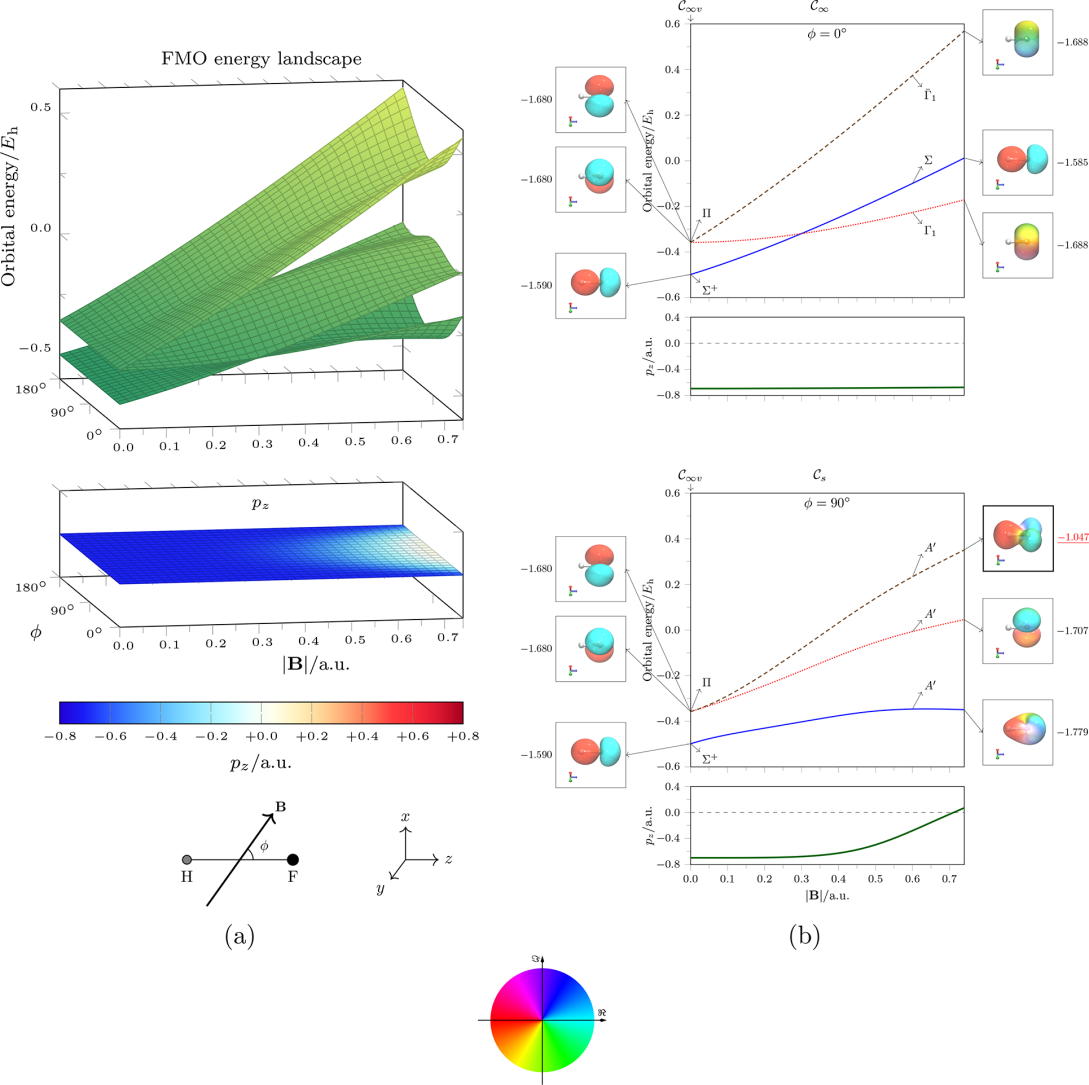
(a) Energy landscapes of the frontier *m*_*s*_ = +1/2 MOs in hydrogen fluoride at various magnetic field strengths and angles. (b) Cross sections through these landscapes at parallel (top) and perpendicular (bottom) field orientations. Annotated on these cross sections are the MO symmetries and isosurfaces plotted at |χ(**r**)| = 0.100. The color at each point **r** on an isosurface indicates the value of arg χ(**r**) at that point according to the accompanying color wheel. The numerical value next to each isosurface gives the value of the orbital electronic dipole moment ⟨χ|μ̂_*z*_|χ⟩ for the associated MO. In , the one-dimensional irreducible representation Γ_1_ has character function χ^Γ_1_^[*Ĉ*_∞_(ϕ)] = exp(*iϕ*), and the corresponding complex-conjugate one-dimensional irreducible representation Γ̅_1_ has character function χ^Γ̅_1_^[*Ĉ*_∞_(ϕ)] = exp(−*iϕ*), where *Ĉ*_∞_(ϕ) denotes an anticlockwise rotation through an angle ϕ as viewed down the *z*-axis.

Figure 7a shows the landscapes of the *m*_*s*_ = +1/2 frontier MOs in hydrogen fluoride (cf. Figure 6) at various strengths and orientations of the external magnetic field together with the values of the electric dipole moment component along the internuclear axis, *p*_*z*_. The H–F bond length is kept fixed at its zero-field equilibrium value, 0.92897 Å, for all field strengths and orientations. It can be seen that, in the region where *p*_*z*_ becomes less negative and approaches zero (|**B**| ≥ 0.5 *B*_0_ ≈ 1.18 × 10^5^ T and ϕ ≈ 90°), the frontier MO landscapes display significant curvature. This suggests that these MOs interact with one another strongly in this region, and these interactions might be responsible for the observed electric dipole reversal. However, in parallel fields, even at very high field strengths (|**B**| ≥ 0.7 *B*_0_ ≈ 1.65 × 10^5^ T), *p*_*z*_ remains at ca. −0.7 au which is approximately the same value as that at zero field. The energy landscapes of the frontier MOs also show very little curvature in the vicinity of ϕ = 0° or 180°, thus implying a lack of interaction between these MOs and further strengthening the conjecture that these MOs must interact in some way to result in a reversal of the electric dipole moment.

In Figure 7b, cross sections through the frontier MO energy landscapes and *p*_*z*_ plots at ϕ = 0° and 90° are shown together with MO symmetry assignments from QSym^2^ and complex isosurface plots as described earlier. It becomes immediately obvious that, at ϕ = 0°, the three frontier MOs have different symmetries at all values of |**B**| and are therefore unable to interact via the KS operator. In fact, the Σ and Γ_1_ energy curves can cross because of their different symmetries. Even though their energies vary quite significantly as |**B**| increases, this variation is only due to the interactions of these MOs with the applied field. Although these interactions do lead to qualitative changes in the shapes of the MOs, most notably the disappearance of nodal planes in the two Π MOs at zero field that become Γ_1_/ Γ̅_1_ MOs at |**B**| > 0, these changes do not actually affect the distribution of electrons along the internuclear axis in any significant way. This is indeed confirmed by the near equality of the ⟨χ|μ̂_*z*_|χ⟩ values of these MOs at |**B**| = 0 and 0.74 *B*_0_. Consequently, the electric dipole moment along the internuclear axis remains almost unchanged.

The situation is markedly different at ϕ = 90°. The three frontier MOs now have the same symmetry in  and are thus permitted to interact via the KS operator. Indeed they do, as is evident from the distortions in their energy curves for  and also in the shapes of their isosurfaces. Most significantly, the highest occupied MO (HOMO) shows the most drastic change from a 2*p* orbital localized entirely on the fluorine atom to a laterally delocalized MO with a pronounced lobe on the hydrogen atom. Associated with this change is the large increase in the value of ⟨χ|μ̂_*z*_|χ⟩ for this MO from −1.680 au at zero field to −1.047 au at |**B**| = 0.74 *B*_0_, which more than outweighs the decreases in the values of ⟨χ|μ̂_*z*_|χ⟩ for the other two frontier MOs. There is thus a partial charge transfer from the fluorine atom to the hydrogen atom in the HOMO induced by the perpendicular magnetic field, which is responsible for the observed dipole reversal.

### 3.4 Symmetry of Electron Densities

In the final set of case studies, we demonstrate the ability of QSym^2^ to perform symmetry analysis for electron densities, as formulated in Section 2.4.2. In particular, we show how the symmetry of the electron density is intimately related to that of the underlying electronic wave function, both at zero field and in the presence of external electric and magnetic fields.

#### 3.4.1 Computational Details

For the above purpose, we chose the equilateral geometry of H_3_^+^ that has been found in ref (37) to be the optimal geometry for the lowest *M*_*S*_ = −1 electronic state when a uniform magnetic field of strength |**B**| = 1.0 *B*_0_ is applied perpendicular to the plane of the molecule. For this geometry, the lowest *M*_*S*_ = −1 wave functions and densities were computed in QUEST(78) at the UHF/6-311++(2+,2+)G** level of theory in three cases: at zero field, in the presence of a perpendicular uniform electric field with strength  au, and in the presence of a perpendicular uniform magnetic field with strength |**B**| = 1.0 *B*_0_. The symmetry assignments for the resulting determinantal wave functions and the corresponding electron densities were then determined by QSym^2^. In all cases, the H_3_^+^ structure was placed in the *yz*-plane so that any external field applied perpendicular to the molecule would be along the *x*-direction.

#### 3.4.2 Density Symmetries in H_3_^+^

Table 5 shows the wave function and density symmetries of the lowest *M*_*S*_ = −1 UHF wave function in the three cases described above. We examine first the perpendicular magnetic field case (labeled *B*_*x*_ in Table 5) where the unitary symmetry group of the molecule-plus-field system is . The lowest *M*_*S*_ = −1 UHF wave function has already been reported in ref (37) to have  symmetry, which is a one-dimensional irreducible representation in  whose character function satisfies χ^Γ′^(*Ĉ*_3_) = exp(2*i*π/3) and χ^Γ′^(σ̂_*h*_) = 1. As this is a nondegenerate wave function, the corresponding density must be totally symmetric in , which is indeed the case as verified by the density symmetry assignment and also by the density isosurface and contours in the *yz*-plane. Here, the electron cloud can be seen to be equidistributed over the three symmetry-equivalent hydrogen nuclei.

**Table 5:**
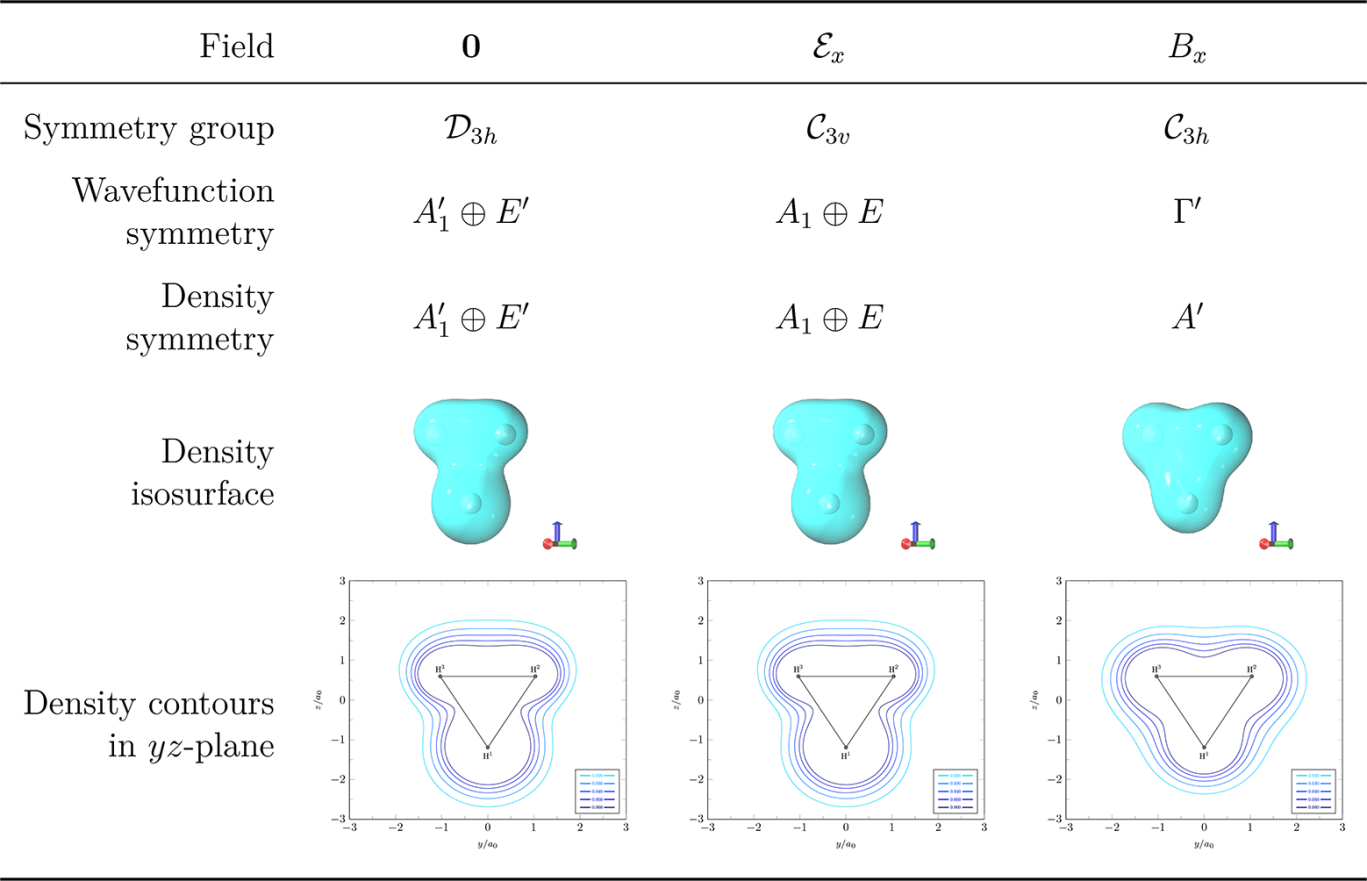
Electronic Wave Function and Total Density Symmetry of the Lowest *M*_*S*_ = −1 state of H_3_^+^ in the Presence of External Electric and Magnetic Fields^a^

a Calculations were performed at the UHF/6-311++(2+,2+)G** level of theory. The magnitude of the applied electric field is 0.1 au, and that of the applied magnetic field is 1.0 *B*_0_. Isosurfaces of total densities are plotted at |ρ(**r**)| = 0.050.

The situation is rather different in the other two cases. In the absence of any external fields (labeled **0** in Table 5), for which the unitary symmetry group is , the lowest *M*_*S*_ = −1 UHF wave function at the same geometry turns out to exhibit symmetry breaking due to its  symmetry. The symmetry analysis of the corresponding total density shows that the density also exhibits an  broken symmetry. Similarly, in the presence of a perpendicular electric field (labeled  in Table 5), for which the unitary symmetry group is reduced to , the UHF wave function now has  symmetry, as does the corresponding density. The symmetry breaking of the total density in these two cases can be visualized most easily via the density isosurfaces and *yz*-contours: the electron cloud is not equally distributed over the three symmetry-equivalent hydrogen nuclei.

The external fields can also be applied parallel to the plane of the molecular frame of H_3_^+^. The wave function and density symmetries and density isosurfaces resulting from the UHF calculations in these field orientations are summarized in Table S1 in the Supporting Information. In all cases, it can be observed that whenever the wave function is nondegenerate, the density is totally symmetric in the prevailing symmetry group, and whenever the wave function exhibits degeneracy, be it because of symmetry breaking or not, the corresponding density is no longer totally symmetric.

We note that it is also possible to obtain density symmetries without having access to any symmetries of the underlying wave function, meaning that the same approach can be readily applied to densities from correlated wave function methods as well as HF or KS-DFT, with no additional implementation. In the context of KS-DFT, this means that symmetry analysis can be carried out directly on the density, rather than the KS orbitals and noninteracting wave function as a proxy for the physical wave function.

Examples of symmetry analyses for KS densities obtained with the r^2^SCAN0 functional are also shown in Table S1 in the Supporting Information for various external field orientations. By considering the symmetries of electron densities, we have a qualitative way to compare HF and KS-DFT calculations: if HF and KS densities have the same symmetry, then there is a likelihood that both calculations describe the same electronic state of the system, but if HF and KS densities differ in their symmetries, then it must be concluded that they are qualitatively different, perhaps because they describe different electronic states, which can happen especially if there are multiple SCF solutions that occur close to one another (see refs^3−5, 25, and 91^ and also Section 3.2). In fact, for the cases listed in Table S1, based on the symmetries of densities, both HF and KS-DFT calculations in each field orientation can be said to approximate the same electronic state.

## 4 Conclusion

A new program for quantum symbolic symmetry analysis, QSym^2^, is presented in this work. A key feature of the program is its capability to generate character tables symbolically on-the-fly, which endows it with the ability to perform symmetry analysis for general groups automatically. This flexibility means that QSym^2^ can yield reliable symmetry assignments for systems exhibiting degeneracy and symmetry breaking effects, where standard implementations cannot be applied. In addition, QSym^2^ can handle reduced symmetries arising in electric or magnetic fields, thus providing a valuable tool for analysis and insight into systems under less chemically intuitive conditions.

The ability of QSym^2^ to perform analysis of high-symmetry systems was demonstrated for C_84_H_64_, C_60_, and B_9_^–^, where in each case, the full molecular symmetry group could be correctly identified and carried through to classify the symmetry of the resulting MOs, including their associated degeneracies. The octahedral transition-metal complex Fe(CN)_6_^3–^ was then used to demonstrate how QSym^2^ is able to deduce representation spaces spanned by symmetry-broken determinants and MOs, giving a way to classify and understand symmetry breaking effects. Furthermore, the capability of QSym^2^ to analyze symmetry in external magnetic fields was demonstrated for the hydrogen fluoride molecule, where the symmetry of the MOs under a magnetic field was shown to provide a rationalization of the behavior of the molecular electric dipole moment as a function of field strength and orientation.

An important benefit of the generic symmetry-orbit-based representation analysis framework formulated in this article and used in QSym^2^ is the ability to analyze symmetry of quantities other than wave functions and MOs that arise in quantum-chemical calculations. As a simple example, the changes in the electron density of the equilateral H_3_^+^ as a function of electric and magnetic field were analyzed, with the density symmetry analysis revealing the symmetry breaking or conservation of the underlying wave function. This approach can be applied on an equal footing to densities arising from SCF calculations or more elaborate post-HF correlated calculations without the need to explicitly perform symmetry analysis on the correlated wave functions.

The generality in the code design of QSym^2^ opens many avenues for future research. In particular, the applicability of the symmetry-orbit-based representation analysis to members of general linear spaces makes it possible to directly consider the symmetry of many important quantities in quantum chemistry. One group of such quantities includes normal coordinates that describe translational, rotational, and vibrational modes of molecules. Another interesting class of such quantities includes functions of electron density and/or density matrix, of which the Fukui function,^105^ which encapsulates chemical reactivity information, and the magnetically induced current density,^106,107^ which provides an interpretation for observations in magnetic spectroscopic methods, are prime examples. Moreover, QSym^2^ can already provide a more complete analysis of magnetic symmetry using corepresentation theory^56,60,62^ and is not limited to uniform external fields—these developments will be reported in future publications.

Finally, we emphasize that the Rust implementation of QSym^2^ is also flexible, such that the program can operate either as a tool to be applied subsequently to a quantum-chemical calculation in a stand-alone manner (as used in this work with Q-Chem), or as a library readily integrated into existing programs (as used in this and earlier^37,108^ work with QUEST(78)). Since the program is open-source, we hope that it will become a useful tool for application in a wide range of chemical simulations.

## Data Availability

The main repository for QSym^2^ can be accessed at https://gitlab.com/bangconghuynh/qsym2 (accessed November 16, 2023). The documentations for QSym^2^ can be found at https://qsym2.dev/ (accessed November 16, 2023). The source code for QSym^2^ v0.7.0 released at the time of publication of this article was deposited permanently at 10.5281/zenodo.8384779 (accessed November 16, 2023).

## Supporting Information Available

Computational design of the SymOp structure, including fields in the SymOp structure, conversion between improper-rotation conventions, equality of SymOp instances, hashability of SymOp instances, and compositability of SymOp instances; algorithms for character table generation including the eigenvalue problem for characters and diagonalization over a finite field; and symmetry of electron densities (PDF)

## References

[ref1] Mjolsness R. C.; Ruppel H. M. Multiple Solutions of Hartree–Fock Equations. J. Comput. Phys. 1968, 3, 259–272. 10.1016/0021-9991(68)90021-1.

[ref2] Fukutome H. Theory of the Unrestricted Hartree–Fock Equation and Its Solutions. I.. Prog. Theor. Phys. 1971, 45, 1382–1406. 10.1143/PTP.45.1382.

[ref3] Stanton R. E. Multiple Solutions to the Hartree–Fock Problem. I. General Treatment of Two-Electron Closed-Shell Systems. J. Chem. Phys. 1968, 48, 257–262. 10.1063/1.1667913.

[ref4] King H. F.; Stanton R. E. Multiple Solutions to the Hartree–Fock Problem. II. Molecular Wavefunctions in the Limit of Infinite Internuclear Separation. J. Chem. Phys. 1969, 50, 3789–3797. 10.1063/1.1671628.

[ref5] Redondo P.; Flores J. R.; Largo-Cabrerizo J. Multiple solutions of unrestricted Hartree–Fock equations: The SNH^+^ radical as an example. J. Comput. Chem. 1989, 10, 295–301. 10.1002/jcc.540100303.

[ref6] Pulay P.; Liu R. F. Methods for Finding Unrestricted Hartree–Fock Solutions and Multiple Solutions. J. Phys. Chem. 1990, 94, 5548–5551. 10.1021/j100377a026.

[ref7] Szabo A.; Ostlund N. S. Modern Quantum Chemistry: Introduction to Advanced Electronic Structure Theory; Dover Publications, Inc.: Mineola, NY, 1996.

[ref8] Parr R. G.; Yang W. Density-Functional Theory of Atoms and Molecules; Oxford University Press: 1989.

[ref9] Nesbet R. K. Configuration Interaction in Orbital Theories. Proc. R. Soc. London Ser. Math. Phys. Sci. 1955, 230, 312–321. 10.1098/rspa.1955.0134.

[ref10] Shavitt I.; Bartlett R. J. Many-Body Methods in Chemistry and Physics; Cambridge University Press: New York, United States, 2009.

[ref11] Helgaker T.; Jørgensen P.; Olsen J. Molecular Electronic-Structure Theory; John Wiley & Sons, Ltd.: Chichester, UK, 2000.

[ref12] Olsen J. CASSCF Method: A Perspective and Commentary. Int. J. Quantum Chem. 2011, 111, 3267–3272. 10.1002/qua.23107.

[ref13] Baerends E. J.; Gritsenko O. V. A Quantum Chemical View of Density Functional Theory. J. Phys. Chem. A 1997, 101, 5383–5403. 10.1021/jp9703768.

[ref14] Stowasser R.; Hoffmann R. What Do the Kohn–Sham Orbitals and Eigenvalues Mean?. J. Am. Chem. Soc. 1999, 121, 3414–3420. 10.1021/ja9826892.

[ref15] Teale A. M.; Helgaker T.; Savin A.; Adamo C.; Aradi B.; Arbuznikov A. V.; Ayers P. W.; Baerends E. J.; Barone V.; Calaminici P.; Cancès E.; Carter E. A.; Chattaraj P. K.; Chermette H.; Ciofini I.; Crawford T. D.; De Proft F.; Dobson J. F.; Draxl C.; Frauenheim T.; Fromager E.; Fuentealba P.; Gagliardi L.; Galli G.; Gao J.; Geerlings P.; Gidopoulos N.; Gill P. M. W.; Gori-Giorgi P.; Görling A.; Gould T.; Grimme S.; Gritsenko O.; Jensen H. J. A.; Johnson E. R.; Jones R. O.; Kaupp M.; Köster A. M.; Kronik L.; Krylov A. I.; Kvaal S.; Laestadius A.; Levy M.; Lewin M.; Liu S.; Loos P.-F.; Maitra N. T.; Neese F.; Perdew J. P.; Pernal K.; Pernot P.; Piecuch P.; Rebolini E.; Reining L.; Romaniello P.; Ruzsinszky A.; Salahub D. R.; Scheffler M.; Schwerdtfeger P.; Staroverov V. N.; Sun J.; Tellgren E.; Tozer D. J.; Trickey S. B.; Ullrich C. A.; Vela A.; Vignale G.; Wesolowski T. A.; Xu X.; Yang W. DFT Exchange: Sharing Perspectives on the Workhorse of Quantum Chemistry and Materials Science. Phys. Chem. Chem. Phys. 2022, 24, 28700–28781. 10.1039/D2CP02827A. 36269074 PMC9728646

[ref16] Levy M.; Perdew J. P.; Sahni V. Exact Differential Equation for the Density and Ionization Energy of a Many-Particle System. Phys. Rev. A 1984, 30, 2745–2748. 10.1103/PhysRevA.30.2745.

[ref17] Savin A.; Umrigar C. J.; Gonze X. Relationship of Kohn–Sham Eigenvalues to Excitation Energies. Chem. Phys. Lett. 1998, 288, 391–395. 10.1016/S0009-2614(98)00316-9.

[ref18] Rubio M.; Roos B. O.; Serrano-Andrés L.; Merchán M. Theoretical Study of the Electronic Spectrum of Magnesium-Porphyrin. J. Chem. Phys. 1999, 110, 7202–7209. 10.1063/1.478624.

[ref19] Rubio M.; Merchán M.; Pou-AméRigo R.; Ortí E. The Low-Lying Excited States of 2,2′-Bithiophene: A Theoretical Analysis. ChemPhysChem 2003, 4, 1308–1315. 10.1002/cphc.200300790. 14714378

[ref20] Tran L. N.; Shea J. A. R.; Neuscamman E. Tracking Excited States in Wave Function Optimization Using Density Matrices and Variational Principles. J. Chem. Theory Comput. 2019, 15, 4790–4803. 10.1021/acs.jctc.9b00351. 31393725

[ref21] Tran L. N.; Neuscamman E. Improving Excited-State Potential Energy Surfaces via Optimal Orbital Shapes. J. Phys. Chem. A 2020, 124, 8273–8279. 10.1021/acs.jpca.0c07593. 32885970

[ref22] Hanscam R.; Neuscamman E. Applying Generalized Variational Principles to Excited-State-Specific Complete Active Space Self-consistent Field Theory. J. Chem. Theory Comput. 2022, 18, 6608–6621. 10.1021/acs.jctc.2c00639. 36215108

[ref23] Jackels C. F.; Davidson E. R. The Two Lowest Energy ^2^*A*′ States of NO_2_. J. Chem. Phys. 1976, 64, 2908–2917. 10.1063/1.432552.

[ref24] Sundstrom E. J.; Head-Gordon M. Non-Orthogonal Configuration Interaction for the Calculation of Multielectron Excited States. J. Chem. Phys. 2014, 140, 114103 10.1063/1.4868120. 24655168

[ref25] Huynh B. C.; Thom A. J. W. Symmetry in Multiple Self-Consistent-Field Solutions of Transition-Metal Complexes. J. Chem. Theory Comput. 2020, 16, 904–930. 10.1021/acs.jctc.9b00900. 31820967

[ref26] Mayhall N. J.; Horn P. R.; Sundstrom E. J.; Head-Gordon M. Spin-Flip Non-Orthogonal Configuration Interaction: A Variational and Almost Black-Box Method for Describing Strongly Correlated Molecules. Phys. Chem. Chem. Phys. 2014, 16, 22694–22705. 10.1039/C4CP02818J. 25233435

[ref27] Görling A. Symmetry in Density-Functional Theory. Phys. Rev. A 1993, 47, 2783–2799. 10.1103/PhysRevA.47.2783. 9909246

[ref28] Görling A. Proper Treatment of Symmetries and Excited States in a Computationally Tractable Kohn-Sham Method. Phys. Rev. Lett. 2000, 85, 4229–4232. 10.1103/PhysRevLett.85.4229. 11060605

[ref29] Savin A. In Theoretical and Computational Chemistry; Seminario J. M., Ed.; Recent Developments and Applications of Modern Density Functional Theory; Elsevier: 1996; Vol. 4, pp 327–357.

[ref30] Chowdhury S. T. U. R.; Perdew J. P. Spherical vs Non-Spherical and Symmetry-Preserving vs Symmetry-Breaking Densities of Open-Shell Atoms in Density Functional Theory. J. Chem. Phys. 2021, 155, 234110 10.1063/5.0072020. 34937366

[ref31] Bersuker I. B. In The Jahn–Teller Effect: Fundamentals and Implications for Physics and Chemistry; Köppel H., Yarkony D. R., Barentzen H., Eds.; Springer Series in Chemical Physics; Springer: Berlin, Heidelberg, 2009; pp 3–23.

[ref32] Ceulemans A. J. Group Theory Applied to Chemistry; Springer Science & Business Media: 2013.

[ref33] Wibowo M.; Irons T. J. P.; Teale A. M. Modeling Ultrafast Electron Dynamics in Strong Magnetic Fields Using Real-Time Time-Dependent Electronic Structure Methods. J. Chem. Theory Comput. 2021, 17, 2137–2165. 10.1021/acs.jctc.0c01269. 33724806 PMC8047917

[ref34] Irons T. J. P.; Huynh B. C.; Teale A. M.; De Proft F.; Geerlings P. Molecular Charge Distributions in Strong Magnetic Fields: A Conceptual and Current DFT Study. Mol. Phys. 2022, e2145245 10.1080/00268976.2022.2145245.

[ref35] Bischoff F. A. Structure of the H_3_ molecule in a strong homogeneous magnetic field as computed by the Hartree–Fock method using multiresolution analysis. Phys. Rev. A 2020, 101, 053413 10.1103/PhysRevA.101.053413.

[ref36] Irons T. J. P.; David G.; Teale A. M. Optimizing Molecular Geometries in Strong Magnetic Fields. J. Chem. Theory Comput. 2021, 17, 2166–2185. 10.1021/acs.jctc.0c01297. 33724812 PMC8047810

[ref37] Wibowo M.; Huynh B. C.; Cheng C. Y.; Irons T. J. P.; Teale A. M. Understanding Ground and Excited-State Molecular Structure in Strong Magnetic Fields Using the Maximum Overlap Method. Mol. Phys. 2023, 121, e2152748 10.1080/00268976.2022.2152748.

[ref38] Epifanovsky E.; Gilbert A. T. B.; Feng X.; Lee J.; Mao Y.; Mardirossian N.; Pokhilko P.; White A. F.; Coons M. P.; Dempwolff A. L.; Gan Z.; Hait D.; Horn P. R.; Jacobson L. D.; Kaliman I.; Kussmann J.; Lange A. W.; Lao K. U.; Levine D. S.; Liu J.; McKenzie S. C.; Morrison A. F.; Nanda K. D.; Plasser F.; Rehn D. R.; Vidal M. L.; You Z.-Q.; Zhu Y.; Alam B.; Albrecht B. J.; Aldossary A.; Alguire E.; Andersen J. H.; Athavale V.; Barton D.; Begam K.; Behn A.; Bellonzi N.; Bernard Y. A.; Berquist E. J.; Burton H. G. A.; Carreras A.; Carter-Fenk K.; Chakraborty R.; Chien A. D.; Closser K. D.; Cofer-Shabica V.; Dasgupta S.; de Wergifosse M.; Deng J.; Diedenhofen M.; Do H.; Ehlert S.; Fang P.-T.; Fatehi S.; Feng Q.; Friedhoff T.; Gayvert J.; Ge Q.; Gidofalvi G.; Goldey M.; Gomes J.; González-Espinoza C. E.; Gulania S.; Gunina A. O.; Hanson-Heine M. W. D.; Harbach P. H. P.; Hauser A.; Herbst M. F.; Hernández Vera M.; Hodecker M.; Holden Z. C.; Houck S.; Huang X.; Hui K.; Huynh B. C.; Ivanov M.; Jász Á.; Ji H.; Jiang H.; Kaduk B.; Kähler S.; Khistyaev K.; Kim J.; Kis G.; Klunzinger P.; Koczor-Benda Z.; Koh J. H.; Kosenkov D.; Koulias L.; Kowalczyk T.; Krauter C. M.; Kue K.; Kunitsa A.; Kus T.; Ladjánszki I.; Landau A.; Lawler K. V.; Lefrancois D.; Lehtola S.; Li R. R.; Li Y.-P.; Liang J.; Liebenthal M.; Lin H.-H.; Lin Y.-S.; Liu F.; Liu K.-Y.; Loipersberger M.; Luenser A.; Manjanath A.; Manohar P.; Mansoor E.; Manzer S. F.; Mao S.-P.; Marenich A. V.; Markovich T.; Mason S.; Maurer S. A.; McLaughlin P. F.; Menger M. F. S. J.; Mewes J.-M.; Mewes S. A.; Morgante P.; Mullinax J. W.; Oosterbaan K. J.; Paran G.; Paul A. C.; Paul S. K.; Pavošević F.; Pei Z.; Prager S.; Proynov E. I.; Rák Á.; Ramos-Cordoba E.; Rana B.; Rask A. E.; Rettig A.; Richard R. M.; Rob F.; Rossomme E.; Scheele T.; Scheurer M.; Schneider M.; Sergueev N.; Sharada S. M.; Skomorowski W.; Small D. W.; Stein C. J.; Su Y.-C.; Sundstrom E. J.; Tao Z.; Thirman J.; Tornai G. J.; Tsuchimochi T.; Tubman N. M.; Veccham S. P.; Vydrov O.; Wenzel J.; Witte J.; Yamada A.; Yao K.; Yeganeh S.; Yost S. R.; Zech A.; Zhang I. Y.; Zhang X.; Zhang Y.; Zuev D.; Aspuru-Guzik A.; Bell A. T.; Besley N. A.; Bravaya K. B.; Brooks B. R.; Casanova D.; Chai J.-D.; Co-riani S.; Cramer C. J.; Cserey G.; DePrince A. E.; DiStasio R. A.; Dreuw A.; Dunietz B. D.; Furlani T. R.; Goddard W. A.; Hammes-Schiffer S.; Head-Gordon T.; Hehre W. J.; Hsu C.-P.; Jagau T.-C.; Jung Y.; Klamt A.; Kong J.; Lambrecht D. S.; Liang W.; Mayhall N. J.; McCurdy C. W.; Neaton J. B.; Ochsenfeld C.; Parkhill J. A.; Peverati R.; Rassolov V. A.; Shao Y.; Slipchenko L. V.; Stauch T.; Steele R. P.; Subotnik J. E.; Thom A. J. W.; Tkatchenko A.; Truhlar D. G.; Van Voorhis T.; Wesolowski T. A.; Whaley K. B.; Woodcock H. L.; Zimmerman P. M.; Faraji S.; Gill P. M. W.; Head-Gordon M.; Herbert J. M.; Krylov A. I. Software for the Frontiers of Quantum Chemistry: An Overview of Developments in the Q-Chem 5 Package. J. Chem. Phys. 2021, 155, 084801 10.1063/5.0055522. 34470363 PMC9984241

[ref39] Neese F. Software Update: The ORCA Program System—Version 5.0. WIREs Comput. Mol. Sci. 2022, 12, e1606 10.1002/wcms.1606.

[ref40] Sun Q.; Berkelbach T. C.; Blunt N. S.; Booth G. H.; Guo S.; Li Z.; Liu J.; McClain J. D.; Sayfutyarova E. R.; Sharma S.; Wouters S.; Chan G. K.-L. PySCF: The Python-based Simulations of Chemistry Framework. WIREs Comput. Mol. Sci. 2018, 8, e1340 10.1002/wcms.1340.

[ref41] Aidas K.; Angeli C.; Bak K. L.; Bakken V.; Bast R.; Boman L.; Christiansen O.; Cimiraglia R.; Coriani S.; Dahle P.; Dalskov E. K.; Ekstrom U.; Enevoldsen T.; Eriksen J. J.; Ettenhuber P.; Fernandez B.; Ferrighi L.; Fliegl H.; Frediani L.; Hald K.; Halkier A.; Hattig C.; Heiberg H.; Helgaker T.; Hennum A. C.; Hettema H.; Hjertenæs E.; Høst S.; Høyvik I.-M.; Iozzi M. F.; Jansik B.; Jensen H. J. A.; Jonsson D.; Jørgensen P.; Kauczor J.; Kirpekar S.; Kjærgaard T.; Klopper W.; Knecht S.; Kobayashi R.; Koch H.; Kongsted J.; Krapp A.; Kristensen K.; Ligabue A.; Lutnæs O. B.; Melo J. I.; Mikkelsen K. V.; Myhre R. H.; Neiss C.; Nielsen C. B.; Norman P.; Olsen J.; Olsen J. M. H.; Osted A.; Packer M. J.; Pawlowski F.; Pedersen T. B.; Provasi P. F.; Reine S.; Rinkevicius Z.; Ruden T. A.; Ruud K.; Rybkin V. V.; Sałek P.; Samson C. C. M.; de Meras A. S.; Saue T.; Sauer S. P. A.; Schimmelpfennig B.; Sneskov K.; Steindal A. H.; Sylvester-Hvid K. O.; Taylor P. R.; Teale A. M.; Tellgren E. I.; Tew D. P.; Thorvaldsen A. J.; Thøgersen L.; Vahtras O.; Watson M. A.; Wilson D. J. D.; Ziolkowski M.; Agren H. The Dalton Quantum Chemistry Program System. WIREs Comput. Mol. Sci. 2014, 4, 269–284. 10.1002/wcms.1172. PMC4171759 25309629

[ref42] Aquilante F.; Autschbach J.; Baiardi A.; Battaglia S.; Borin V. A.; Chibotaru L. F.; Conti I.; De Vico L.; Delcey M.; Fdez. Galván I.; Ferré N.; Freitag L.; Garavelli M.; Gong X.; Knecht S.; Larsson E. D.; Lindh R.; Lundberg M.; Malmqvist P. Å.; Nenov A.; Norell J.; Odelius M.; Olivucci M.; Pedersen T. B.; Pedraza-González L.; Phung Q. M.; Pierloot K.; Reiher M.; Schapiro I.; Segarra-Martí J.; Segatta F.; Seijo L.; Sen S.; Sergentu D.-C.; Stein C. J.; Ungur L.; Vacher M.; Valentini A.; Veryazov V. Modern Quantum Chemistry with [Open]Molcas. J. Chem. Phys. 2020, 152, 214117 10.1063/5.0004835. 32505150

[ref43] Smith D. G. A.; Burns L. A.; Simmonett A. C.; Parrish R. M.; Schieber M. C.; Galvelis R.; Kraus P.; Kruse H.; Di Remigio R.; Alenaizan A.; James A. M.; Lehtola S.; Misiewicz J. P.; Scheurer M.; Shaw R. A.; Schriber J. B.; Xie Y.; Glick Z. L.; Sirianni D. A.; O’Brien J. S.; Waldrop J. M.; Kumar A.; Hohenstein E. G.; Pritchard B. P.; Brooks B. R.; Schaefer H. F.; Sokolov A. Y.; Patkowski K.; DePrince A. E.; Bozkaya U.; King R. A.; Evangelista F. A.; Turney J. M.; Crawford T. D.; Sherrill C. D. PSI4 1.4: Open-source Software for High-Throughput Quantum Chemistry. J. Chem. Phys. 2020, 152, 184108 10.1063/5.0006002. 32414239 PMC7228781

[ref44] Matthews D. A.; Cheng L.; Harding M. E.; Lipparini F.; Stopkowicz S.; Jagau T.-C.; Szalay P. G.; Gauss J.; Stanton J. F. Coupled-Cluster Techniques for Computational Chemistry: The CFOUR Program Package. J. Chem. Phys. 2020, 152, 214108 10.1063/5.0004837. 32505146

[ref45] Balasubramani S. G.; Chen G. P.; Coriani S.; Diedenhofen M.; Frank M. S.; Franzke Y. J.; Furche F.; Grotjahn R.; Harding M. E.; Hättig C.; Hellweg A.; Helmich-Paris B.; Holzer C.; Huniar U.; Kaupp M.; Marefat Khah A.; Karbalaei Khani S.; Müller T.; Mack F.; Nguyen B. D.; Parker S. M.; Perlt E.; Rappoport D.; Reiter K.; Roy S.; Rückert M.; Schmitz G.; Sierka M.; Tapavicza E.; Tew D. P.; van Wüllen C.; Voora V. K.; Weigend F.; Wodyński A.; Yu J. M. TURBOMOLE: Modular Program Suite for Ab Initio Quantum-Chemical and Condensed-Matter Simulations. J. Chem. Phys. 2020, 152, 184107 10.1063/5.0004635. 32414256 PMC7228783

[ref46] Matsakis N. D.; Klock F. S. The Rust Language. Ada Lett. 2014, 34, 103–104. 10.1145/2692956.2663188.

[ref47] Klabnik S.; Nichols C. The Rust Programming Language; No Starch Press: 2018.

[ref48] Aschi M.; Spezia R.; Di Nola A.; Amadei A. A First-Principles Method to Model Perturbed Electronic Wavefunctions: The Effect of an External Homogeneous Electric Field. Chem. Phys. Lett. 2001, 344, 374–380. 10.1016/S0009-2614(01)00638-8.

[ref49] Weil J. A.; Bolton J. R. Electron Paramagnetic Resonance; John Wiley & Sons, Inc.: Hoboken, NJ, 2007.

[ref50] Tellgren E. I.; Laestadius A.; Helgaker T.; Kvaal S.; Teale A. M. Uniform Magnetic Fields in Density-Functional Theory. J. Chem. Phys. 2018, 148, 024101 10.1063/1.5007300. 29331113

[ref51] Altmann S. L. Rotations, Quaternions, and Double Groups; Dover Publications, Inc.: New York, 2005.

[ref52] Beruski O.; Vidal L. N. Algorithms for Computer Detection of Symmetry Elements in Molecular Systems. J. Comput. Chem. 2014, 35, 290–299. 10.1002/jcc.23493. 24403016

[ref53] Pausch A.; Gebele M.; Klopper W. Molecular Point Groups and Symmetry in External Magnetic Fields. J. Chem. Phys. 2021, 155, 201101 10.1063/5.0069859. 34852467

[ref54] Birss R. R. Symmetry and Magnetism; North-Holland Pub. Co.: Amsterdam, 1966.

[ref55] Dimmock J. O.; Wheeler R. G. Symmetry Properties of Wave Functions in Magnetic Crystals. Phys. Rev. 1962, 127, 391–404. 10.1103/PhysRev.127.391.

[ref56] Bradley C. J.; Davies B. L. Magnetic Groups and Their Corepresentations. Rev. Mod. Phys. 1968, 40, 359–379. 10.1103/RevModPhys.40.359.

[ref57] Lazzeretti P.; Rossi E.; Zanasi R. Singularities of Magnetic-Field Induced Electron Current Density: A Study of the Ethylene Molecule. Int. J. Quantum Chem. 1984, 25, 929–940. 10.1002/qua.560250602.

[ref58] Keith T. A.; Bader R. F. W. Topological Analysis of Magnetically Induced Molecular Current Distributions. J. Chem. Phys. 1993, 99, 3669–3682. 10.1063/1.466165.

[ref59] Pelloni S.; Lazzeretti P. Stagnation Graphs and Topological Models of Magnetic-Field Induced Electron Current Density for Some Small Molecules in Connection with Their Magnetic Symmetry. Int. J. Quantum Chem. 2011, 111, 356–367. 10.1002/qua.22658.

[ref60] Wigner E. Theory and Its Application to the Quantum Mechanics of Atomic Spectra; Academic Press: London, 1959.

[ref61] Cracknell A. P. Corepresentations of Magnetic Cubic Space Groups. Prog. Theor. Phys. 1965, 33, 812–827. 10.1143/PTP.33.812.

[ref62] Cracknell A. P. Corepresentations of Magnetic Point Groups. Prog. Theor. Phys. 1966, 35, 196–213. 10.1143/PTP.35.196.

[ref63] Newmarch J. D.; Golding R. M. The Character Table for the Corepresentations of Magnetic Groups. J. Math. Phys. 1982, 23, 695–704. 10.1063/1.525423.

[ref64] Newmarch J. D. Some Character Theory for Groups of Linear and Antilinear Operators. J. Math. Phys. 1983, 24, 742–756. 10.1063/1.525790.

[ref65] Dixon J. D. High Speed Computation of Group Characters. Numer. Math. 1967, 10, 446–450. 10.1007/BF02162877.

[ref66] Schneider G. J. A. Dixon’s Character Table Algorithm Revisited. J. Symb. Comput. 1990, 9, 601–606. 10.1016/S0747-7171(08)80077-6.

[ref67] Grove L. C. Groups and Characters; John Wiley & Sons, Inc.: New York, United States, 1997.

[ref68] GAP – Groups, Algorithms, and Programming. The GAP Group. 2022. https://www.gap-system.org (accessed November 14, 2023).

[ref69] Mulliken R. S. Report on Notation for the Spectra of Polyatomic Molecules. J. Chem. Phys. 1955, 23, 1997–2011. 10.1063/1.1740655.

[ref70] Fowler P. W.; Gray B. R. Induced Currents and Electron Counting in Aromatic Boron Wheels: B_8_^2–^ and B_9_^–^. Inorg. Chem. 2007, 46, 2892–2897. 10.1021/ic062302t. 17343375

[ref71] Đorđević S.; Solà M.; Radenković S. Aromaticity of Singlet and Triplet Boron Disk-like Clusters: A Test for Electron Counting Aromaticity Rules. Inorg. Chem. 2022, 61, 10116–10125. 10.1021/acs.inorgchem.2c01197. 35737864

[ref72] Karttunen A. J.; Linnolahti M.; Pakkanen T. A. Structural and Electronic Characteristics of Diamondoid Analogues of Group 14 Elements. J. Phys. Chem. C 2008, 112, 16324–16330. 10.1021/jp804695s.

[ref73] Foerster A.; Besley N. A. Quantum Chemical Characterization and Design of Quantum Dots for Sensing Applications. J. Phys. Chem. A 2022, 126, 2899–2908. 10.1021/acs.jpca.2c00947. 35502789 PMC9125561

[ref74] Soriano M.; Palacios J. J. Theory of Projections with Nonorthogonal Basis Sets: Partitioning Techniques and Effective Hamiltonians. Phys. Rev. B 2014, 90, 075128 10.1103/PhysRevB.90.075128.

[ref75] Plasser F.; Ruckenbauer M.; Mai S.; Oppel M.; Marquetand P.; González L. Efficient and Flexible Computation of Many-Electron Wave Function Overlaps. J. Chem. Theory Comput. 2016, 12, 1207–1219. 10.1021/acs.jctc.5b01148. 26854874 PMC4785508

[ref76] Valeev E. F. Libint: A Library for the Evaluation of Molecular Integrals of Many-Body Operators over Gaussian Functions. 2023; http://libint.valeyev.net/ (accessed November 14, 2023).

[ref77] Sun Q. Libcint: An Efficient General Integral Library for Gaussian Basis Functions. J. Comput. Chem. 2015, 36, 1664–1671. 10.1002/jcc.23981. 26123808

[ref78] QUEST, a Rapid Development Platform for QUantum Electronic Structure Techniques. 2022. https://quest.codes (accessed November 14, 2023).

[ref79] Tellgren E. I.; Soncini A.; Helgaker T. Nonperturbative Ab Initio Calculations in Strong Magnetic Fields Using London Orbitals. J. Chem. Phys. 2008, 129, 154114 10.1063/1.2996525. 19045183

[ref80] BAGEL, Brilliantly Advanced General Electronic-structure Library. Available under the GNU General Public License. https://nubakery.org/ (accessed November 14, 2023).

[ref81] Shiozaki T. BAGEL: Brilliantly Advanced General Electronic-structure Library. WIREs Comput. Mol. Sci. 2018, 8, e1331 10.1002/wcms.1331.

[ref82] Williams-Young D. B.; Petrone A.; Sun S.; Stetina T. F.; Lestrange P.; Hoyer C. E.; Nascimento D. R.; Koulias L.; Wildman A.; Kasper J.; Goings J. J.; Ding F.; DePrince A. E.; Valeev E. F.; Li X. The Chronus Quantum Software Package. WIREs Comput. Mol. Sci. 2020, 10, e1436 10.1002/wcms.1436.

[ref83] Honda M.; Sato K.; Obara S. Formulation of Molecular Integrals over Gaussian Functions Treatable by Both the Laplace and Fourier Transforms of Spatial Operators by Using Derivative of Fourier-kernel Multiplied Gaussians. J. Chem. Phys. 1991, 94, 3790–3804. 10.1063/1.459751.

[ref84] Strictly speaking, the integrals obtained from these packages only give the spatial part of the spin-spatial integral in Equation 27. The spin part is typically handled separately and implicitly, especially for the commonly used spin functions α and β whose orthogonality is known.

[ref85] Lieb E. H. Density Functionals for Coulomb Systems. Int. J. Quantum Chem. 1983, 24, 243–277. 10.1002/qua.560240302.

[ref86] Altmann S. L.; Herzig P. Point-Group Theory Tables. 2011. https://phaidra.univie.ac.at/o:104731 (accessed November 14, 2023).

[ref87] Dacre P. D. On the Use of Symmetry in SCF Calculations. Chem. Phys. Lett. 1970, 7, 47–48. 10.1016/0009-2614(70)80244-5.

[ref88] Elder M. Use of Molecular Symmetry in SCF Calculations. Int. J. Quantum Chem. 1973, 7, 75–85. 10.1002/qua.560070109.

[ref89] Pitzer R. M. Contribution of Atomic Orbital Integrals to Symmetry Orbital Integrals. J. Chem. Phys. 1973, 58, 3111–3112. 10.1063/1.1679625.

[ref90] Häser M.; Almlöf J.; Feyereisen M. W. Exploiting Non-Abelian Point Group Symmetry in Direct Two-Electron Integral Transformations. Theoret. Chim. Acta 1991, 79, 115–122. 10.1007/BF01127100.

[ref91] Thom A. J. W.; Head-Gordon M. Locating Multiple Self-Consistent Field Solutions: An Approach Inspired by Metadynamics. Phys. Rev. Lett. 2008, 101, 193001 10.1103/PhysRevLett.101.193001. 19113263

[ref92] Pulay P. Convergence Acceleration of Iterative Sequences. The Case of SCF Iteration. Chem. Phys. Lett. 1980, 73, 393–398. 10.1016/0009-2614(80)80396-4.

[ref93] Pipek J.; Mezey P. G. Dependence of MO Shapes on a Continuous Measure of Delocalization. Int. J. Quantum Chem. 1988, 34, 1–13. 10.1002/qua.560340804.

[ref94] Pipek J.; Mezey P. G. A Fast Intrinsic Localization Procedure Applicable for Ab Initio and Semiempirical Linear Combination of Atomic Orbital Wave Functions. J. Chem. Phys. 1989, 90, 4916–4926. 10.1063/1.456588.

[ref95] Tanabe Y.; Sugano S. On the Absorption Spectra of Complex Ions II. J. Phys. Soc. Jpn. 1954, 9, 766–779. 10.1143/JPSJ.9.766.

[ref96] England W.; Salmon L. S.; Ruedenberg K. Molecular Orbitals; Springer-Verlag: Berlin/Heidelberg, 1971; Vol. 23/1; pp 31–123.

[ref97] Thom A. J. W.; Sundstrom E. J.; Head-Gordon M. LOBA: A Localized Orbital Bonding Analysis to Calculate Oxidation States, with Application to a Model Water Oxidation Catalyst. Phys. Chem. Chem. Phys. 2009, 11, 11297 10.1039/b915364k. 20024398

[ref98] Lykos P.; Pratt G. W. Discussion on The Hartree-Fock Approximation. Rev. Mod. Phys. 1963, 35, 496–501. 10.1103/RevModPhys.35.496.

[ref99] Dunning T. H. Jr. Gaussian Basis Sets for Use in Correlated Molecular Calculations. I. The Atoms Boron through Neon and Hydrogen. J. Chem. Phys. 1989, 90, 1007–1023. 10.1063/1.456153.

[ref100] Wilson A. K.; Woon D. E.; Peterson K. A.; Dunning T. H. Jr. Gaussian Basis Sets for Use in Correlated Molecular Calculations. IX. The Atoms Gallium through Krypton. J. Chem. Phys. 1999, 110, 7667–7676. 10.1063/1.478678.

[ref101] Stoychev G. L.; Auer A. A.; Neese F. Automatic Generation of Auxiliary Basis Sets. J. Chem. Theory Comput. 2017, 13, 554–562. 10.1021/acs.jctc.6b01041. 28005364

[ref102] This choice is merely for computational convenience because symmetry properties of MOs must be gauge-origin-invariant.

[ref103] Humphrey W.; Dalke A.; Schulten K. VMD: Visual Molecular Dynamics. J. Mol. Graph. 1996, 14, 33–38. 10.1016/0263-7855(96)00018-5. 8744570

[ref104] Al-Saadon R.; Shiozaki T.; Knizia G. Visualizing Complex-Valued Molecular Orbitals. J. Phys. Chem. A 2019, 123, 3223–3228. 10.1021/acs.jpca.9b01134. 30900892

[ref105] Ayers P. W.; Yang W.; Bartolotti L. J. Chemical Reactivity Theory – A Density Functional View; CRC Press: 2009.

[ref106] Sundholm D.; Fliegl H.; Berger R. J. F. Calculations of Magnetically Induced Current Densities: Theory and Applications. WIREs Comput. Mol. Sci. 2016, 6, 639–678. 10.1002/wcms.1270.

[ref107] Sundholm D.; Dimitrova M.; Berger R. J. F. Current Density and Molecular Magnetic Properties. Chem. Commun. 2021, 57, 12362–12378. 10.1039/D1CC03350F. 34726205

[ref108] Wibowo-Teale M.; Ennifer B. J.; Wibowo-Teale A. M. Real-Time Time-Dependent Self-Consistent Field Methods with Dynamic Magnetic Fields. J. Chem. Phys. 2023, 159, 104102 10.1063/5.0160317. 37681694

