# Supplementary material for: QSym2: A Quantum Symbolic Symmetry Analysis Program for Electronic Structure
Source: J Chem Theory Comput. 2023 Dec 25;20(1):114–33. doi: 10.1021/acs.jctc.3c01118 (PMC10782455; doi:10.1021/acs.jctc.3c01118)
Supplement: Supplementary file 1 — ct3c01118_si_001.pdf [file ct3c01118_si_001.pdf]

Supporting Information: QSym²: A Quantum Symbolic Symmetry Analysis Program for Electronic Structure

Bang C. Huynh,* Meilani Wibowo-Teale, and Andrew M. Wibowo-Teale

School of Chemistry, University of Nottingham, Nottingham NG7 2RD, United Kingdom

E-mail: bang.huynh@nottingham.ac.uk

Contents

S1 Computational design of the `SymOp` structure **S-2**

S1.1 Fields in the `SymOp` structure S-2

S1.2 Conversion between improper-rotation conventions S-3

S1.3 Equality of `SymOp` instances S-6

S1.4 Hashability of `SymOp` instances S-7

S1.5 Compositability of `SymOp` instances S-8

S2 Algorithms for character table generation **S-9**

S2.1 The eigenvalue problem for characters S-9

S2.2 Diagonalization over a finite field S-11

S3 Symmetry of electron densities **S-12**

References **S-13**

S1 Computational design of the `SymOp` structure

S1.1 Fields in the `SymOp` structure

Every finite-order symmetry operation $\hat{g}$ of molecular systems in three dimensions can be written most generally as

$$\hat{g} = \hat{t}\hat{s}\hat{C}_n^k(\mathbf{n}). \tag{S-1}$$

In the above expression, $\hat{C}_n^k(\mathbf{n})$ denotes a proper rotation about an axis defined by the normalized vector $\mathbf{n}$ through an angle $2\pi k/n$ as viewed down $\mathbf{n}$ where the positive sign is associated with the *anticlockwise* direction. It must be noted that both k and n are constrained to be integers such that

$$n \geq 1, \quad \left\lfloor -\frac{n}{2} \right\rfloor < k \leq \left\lfloor \frac{n}{2} \right\rfloor, \quad \text{and} \quad \gcd(k, n) = 1 \text{ if } k \neq 0, \tag{S-2}$$

so that all possible rotation angles can be uniquely represented over the rationals via the irreducible fractions

$$\frac{k}{n} \in \left(-\frac{1}{2}, \frac{1}{2} \right] \cap \mathbb{Q}. \tag{S-3}$$

This choice of $k \in \mathbb{Z}/n\mathbb{Z}$ ensures that the identity operation, which corresponds to $k = 0$, always lies at the middle of the interval. Furthermore, the restriction of rotation angle fractions from the reals to the rationals over $(-1/2, 1/2]$ is possible because of the discrete nature of atomic arrangements in molecular systems.

The proper rotation $\hat{C}_n^k(\mathbf{n})$ may then be accompanied by a potentially improper involution:

$$\hat{s} = \begin{cases} \hat{e} & \text{if } \hat{g} \text{ is proper,} \\ \hat{i} & \text{if } \hat{g} \text{ is an inversion-rotation,} \\ \hat{\sigma}_{\mathbf{n}} & \text{if } \hat{g} \text{ is a reflection-rotation,} \end{cases} \tag{S-4}$$

where $\hat{e}$ is the identity, $\hat{i}$ the spatial inversion, and $\hat{\sigma}_{\mathbf{n}}$ the reflection in a plane perpendicular

to $\mathbf{n}$. If $\hat{s} = \hat{\sigma}_{\mathbf{n}}$, then the composition $\hat{\sigma}_{\mathbf{n}}\hat{C}_n^k$ is commonly written as $\hat{S}_n^k$ where care must be taken when interpreting the exponent k which belongs only to the proper-rotation part of the composition. $\hat{C}_n^k(\mathbf{n})$ may also be accompanied by a potentially antiunitary involution:

$$\hat{t} = \begin{cases} \hat{e} & \text{if } \hat{g} \text{ is unitary,} \\ \hat{\theta} & \text{if } \hat{g} \text{ is antiunitary,} \end{cases} \tag{S-5}$$

where $\hat{\theta}$ is the time-reversal operation introduced in Section 2.1.3 of the main text.

To faithfully represent any symmetry operation with the general form shown in Equation (S-1), the `SymOp` structure is, in its simplest form, a product type over the following constituent fields:

(i) `t`: an enumerated type representing $\hat{t}$ whose possible variants are `identity` for $\hat{e}$ or `time_reversal` for $\hat{\theta}$,

(ii) `s`: an enumerated type representing $\hat{s}$ whose possible variants are `identity` for $\hat{e}$, `inversion` for $\hat{i}$, or `reflection` for $\hat{\sigma}_{\mathbf{n}}$,

(iii) `frac`: an ordered pair of integers representing the irreducible fraction k/n, and

(iv) `axis`: an ordered triplet of floating-point numbers representing the three components of $\mathbf{n}$.

S1.2 Conversion between improper-rotation conventions

Both forms of improper rotations are supported in QSym^2 [Equation (S-4)] since reflection-rotations are more common in chemical applications of symmetry,[S1] whereas inversion-rotations are much more natural for comparisons and compositions of symmetry operations.[S2] It is therefore essential to be able to convert between the two forms of any improper

rotation. In other words, given $\hat{\sigma}_{\mathbf{n}}\hat{C}_n^k(\mathbf{n})$, we seek integers k' and n' such that

$$\hat{\sigma}_{\mathbf{n}}\hat{C}_n^k(\mathbf{n}) = \hat{i}\hat{C}_{n'}^{k'}(\mathbf{n}), \tag{S-6}$$

where

$$n' \geq 1, \quad \left\lfloor -\frac{n'}{2} \right\rfloor < k' \leq \left\lfloor \frac{n'}{2} \right\rfloor, \quad \text{and} \quad \gcd(k', n') = 1 \text{ if } k' \neq 0,$$

just as k and n in Equation (S-2). By recognising that $\hat{\sigma}_{\mathbf{n}} = \hat{i}\hat{C}_2(\mathbf{n})$, we deduce from Equation (S-6) the relation

$$\hat{C}_2(\mathbf{n})\hat{C}_n^k(\mathbf{n}) = \hat{C}_{n'}^{k'}(\mathbf{n}), \tag{S-7}$$

which is represented on the parametric ball of $\mathsf{SO}(3)$ rotations in Figure S1 (*cf.* Chapter 10 of Ref. S2 for a detailed description of the rotation parametric ball). It is clear from Figure S1 that this relation only holds when k and k' are both non-zero and have opposite signs, and that the following equation constraining k, k', n, and n' must be true:

$$\frac{2\pi k}{n} - \frac{2\pi k'}{n'} = \begin{cases} \pi & k > 0, k' < 0 \\ -\pi & k < 0, k' > 0 \end{cases} \tag{S-8}$$

We consider first the case $k > 0, k' < 0$. Equation (S-8) becomes

$$\frac{2k}{n} - \frac{2k'}{n'} = 1 \implies n' = \frac{2n}{\frac{n-2k}{-k'}}.$$

Let $x = \frac{n-2k}{-k'}$. For n' to be a positive integer, x must also be a positive integer such that $x \mid 2n$ and $x \mid (n-2k)$. If n' is to be the smallest possible solution, then x must be the largest integer possible that satisfies the above requirements. Thus, $x = \gcd(2n, n-2k)$, and consequently,

$$\begin{aligned} k' &= -\frac{n-2k}{\gcd(2n, n-2k)}, \\ n' &= \frac{2n}{\gcd(2n, n-2k)}. \end{aligned} \qquad (k > 0, k' < 0)$$

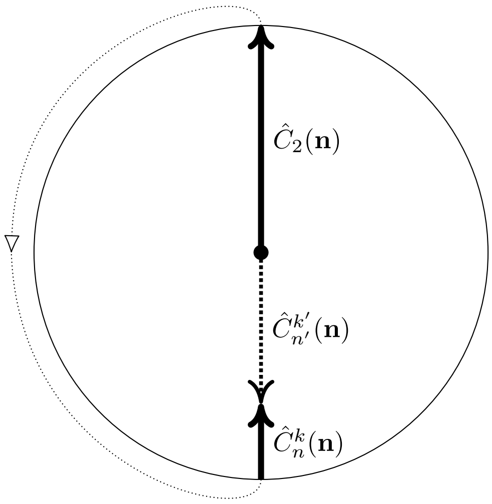

Figure S1: The relation $\hat{C}_2(\mathbf{n})\hat{C}_n^k(\mathbf{n}) = \hat{C}_{n'}^{k'}(\mathbf{n})$ represented on the parametric ball of rotations in $\mathsf{SO}(3)$. The commutativity between $\hat{C}_2(\mathbf{n})$ and $\hat{C}_n^k(\mathbf{n})$ has been exploited to place the path for the $\hat{C}_2(\mathbf{n})$ rotation in a more convenient location.

For the second case where $k < 0, k' > 0$, by a similar argument, we obtain

$$\begin{aligned} k' &= \frac{n+2k}{\gcd(2n, n+2k)}, \\ n' &= \frac{2n}{\gcd(2n, n+2k)}. \end{aligned} \qquad (k < 0, k' > 0)$$

The two cases can then be combined to yield a single set of equations giving k' and n' in terms of k and n by

$$\begin{aligned} k' &= \frac{(2|k| - n)\operatorname{sgn} k}{\gcd(2n, n-2|k|)}, \\ n' &= \frac{2n}{\gcd(2n, n-2|k|)}. \end{aligned} \tag{S-9}$$

Furthermore, the conversion in Equation (S-6) is self-inverse, so if given $\hat{i}\hat{C}_{n'}^{k'}(\mathbf{n})$, one can obtain the values of k and n in $\hat{\sigma}_{\mathbf{n}}\hat{C}_n^k(\mathbf{n})$ using the expressions in Equation (S-9) but with the primed and unprimed quantities swapped.

S1.3 Equality of `SymOp` instances

The 2π-periodicity of spatial proper rotations must be accounted for by the equality comparisons of `SymOp` instances. In particular, this periodicity means that $\hat{C}_n^k(\mathbf{n}) = \hat{C}_n^{-k}(-\mathbf{n})$. To this end, the concept of rotation poles introduced in Chapter 9 of Ref. S2 is utilized where a unique point on the unit sphere in three dimensions is defined for every rotation. The rotation pole for a proper rotation $\hat{C}_n^k(\mathbf{n})$ implemented in QSYM^2 is given by

$$\Pi[\hat{C}_n^k(\mathbf{n})] = \begin{cases} \mathbf{n} & \text{if } 0 < k < n/2, \\ \mathbf{n}_+ & \text{if } n \text{ is even and } k = n/2, \\ -\mathbf{n} & \text{if } k < 0, \\ \mathbf{z} & \text{if } k = 0, \end{cases}$$

where $\mathbf{z}$ is the Cartesian unit vector along the z-direction and $\mathbf{n}_+$ denotes whichever of $\mathbf{n}$ or $-\mathbf{n}$ that lies on a predefined positive unit hemisphere. The *standard* positive unit hemisphere[S2] contains all points that satisfy any one of the following conditions:

(i) $n_z > 0$; or

(ii) $n_z = 0, n_x > 0$; or

(iii) $n_z = 0, n_x = 0, n_y > 0$.

However, other definitions of positive unit hemispheres, including those in which the hemisphere consists of non-adjacent spherical wedges, are possible. Geometrically, one interprets the rotation pole of $\hat{C}_n^k(\mathbf{n})$ as the point on the unit sphere that is left invariant by $\hat{C}_n^k(\mathbf{n})$ and such that this rotation appears as anticlockwise through a positive angle $\phi = \frac{2\pi|k|}{n} \in (0, \pi]$ when observed from outside the sphere (Figure S2).[S2] Thus, comparing $\hat{C}_n^k(\mathbf{n})$ operations where $k \neq 0$ is equivalent to comparing their poles $\Pi[\hat{C}_n^k(\mathbf{n})]$ and positive angles ϕ. For $k = 0$, $\hat{C}_n^0(\mathbf{n})$ is simply the identity, irrespective of the values of n and $\mathbf{n}$, and so all $\hat{C}_n^0(\mathbf{n})$ must be regarded as identical.

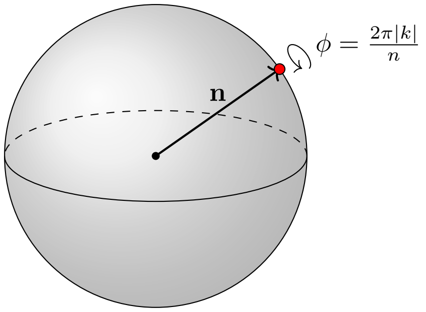

Figure S2: The rotation pole and positive rotation angle of the rotation $\hat{C}_n^k(\mathbf{n})$.

It should also be noted that any improper rotation must be converted to the inversion-rotation form prior to equality comparisons, so that the pole of $\hat{i}\hat{C}_n^k(\mathbf{n})$ can be identified with that of $\hat{C}_n^k(\mathbf{n})$, as shown in Chapter 9 of Ref. S2. Once this has been ensured, two symmetry operations $\hat{g} = \hat{t}\hat{s}\hat{C}_n^k(\mathbf{n})$ and $\hat{g}' = \hat{t}'\hat{s}'\hat{C}_{n'}^{k'}(\mathbf{n}')$ can easily be compared term-wise, where the comparison of $\hat{C}_n^k(\mathbf{n})$ and $\hat{C}_{n'}^{k'}(\mathbf{n}')$ is achieved via their poles and positive angles as described above. Consequently, the comparison of the corresponding `SymOp`$(\hat{g})$ and `SymOp`$(\hat{g}')$ instances is effected through comparisons of the `t` and `s` fields and of the poles and positive rotation angles calculated from the `frac` and `axis` fields.

S1.4 Hashability of `SymOp` instances

In light of the above discussions, an equality-compatible hashing algorithm on the `SymOp` structure must take several factors into account. First, spatial improper rotations must be converted to the inversion-rotation form prior to hashing to ensure that the same improper rotation in whichever form is hashed to the same value. Then, the fields `t` and `s` must constitute two independent arguments in the hash function, since unitary and antiunitary operations must be hashed to different values, as must proper and improper operations. Finally, the `frac` and `axis` fields are used to compute the pole and positive rotation angle which then constitute two additional arguments in the hash function, while making sure that the identity is hashed uniquely.

Vectors whose components are real numbers constitute the `axis` field and thus require

a careful treatment in the comparison and hashing of `SymOp`, since comparing and hashing real numbers is normally discouraged because of the potential violation of transitivity. For instance, given a threshold ϵ, two real vectors $\mathbf{u}$ and $\mathbf{v}$ are considered to be equal with respect to this threshold if $\|\mathbf{u}-\mathbf{v}\| \leq \epsilon$. However, if there is another real vector $\mathbf{w}$ such that $\|\mathbf{v}-\mathbf{w}\| \leq \epsilon$, but that $\|\mathbf{u}-\mathbf{w}\| > \epsilon$, then there is ambiguity in ascertaining the equality between $\mathbf{u}$ and $\mathbf{w}$ with respect to the same ϵ.

Fortunately, owing to the discrete nature of molecular symmetry, suitable choices for ϵ are possible whereby the above ambiguity is avoided when comparing and hashing poles of `SymOp`. In particular, if one chooses any $\epsilon < d_{\mathrm{min}}/2$ where d_{min} is the minimum distance between any two poles of all symmetry operations in the system, then the ϵ-neighborhoods[S3] of symmetry poles are all disjoint. Then, all vectors within the same ϵ-neighborhood of a pole are considered equal to one another and hashed to the same value.

S1.5 Compositability of `SymOp` instances

Let $\hat{g} = \hat{t}\hat{s}\hat{C}_n^k(\mathbf{n})$ and $\hat{g}' = \hat{t}'\hat{s}'\hat{C}_{n'}^{k'}(\mathbf{n}')$ be two symmetry operations. It can be seen from Equation (S-5) that the potentially antiunitary operator $\hat{t}$ always commutes with the spatial unitary operators $\hat{s}$ and $\hat{C}_n^k(\mathbf{n})$ since $\hat{t} = \hat{\theta}$ only acts on spin coordinates. However, the potentially improper operator $\hat{s}$ need not commute with $\hat{C}_n^k(\mathbf{n})$ because reflections and rotations in general do not commute. Fortunately, spatial inversion does commute with rotations, so commutativity between $\hat{s}$ and $\hat{C}_n^k(\mathbf{n})$ can be guaranteed by ensuring that $\hat{g}$ is expressed as an inversion-rotation if it is an improper operation. With these commutativity conditions satisfied, the composition between $\hat{g}$ and $\hat{g}'$ is given by

$$\hat{g}\hat{g}' = (\hat{t}\hat{t}')(\hat{s}\hat{s}')\left[\hat{C}_n^k(\mathbf{n})\hat{C}_{n'}^{k'}(\mathbf{n}')\right].$$

The compositions $\hat{t}\hat{t}'$ and $\hat{s}\hat{s}'$ are trivial to compute. On the other hand, the rotation composition $\hat{C}_n^k(\mathbf{n})\hat{C}_{n'}^{k'}(\mathbf{n}')$ is handled via quaternion algebra[S2] in which each rotation is represented

by a normalized quaternion:

$$\hat{C}_n^k(\mathbf{n}) \equiv [\![\lambda, \boldsymbol{\Lambda}]\!],$$

where

$$\lambda = \cos\frac{k\pi}{n}, \quad \boldsymbol{\Lambda} = \mathbf{n}\sin\frac{k\pi}{n}.$$

The composition can then be computed algebraically:

$$\begin{aligned}\hat{C}_n^k(\mathbf{n})\hat{C}_{n'}^{k'}(\mathbf{n}') &\equiv [\![\lambda, \boldsymbol{\Lambda}]\!][\![\lambda', \boldsymbol{\Lambda}']\!] \\ &= [\![\lambda\lambda' - \boldsymbol{\Lambda}\cdot\boldsymbol{\Lambda}', \lambda\boldsymbol{\Lambda}' + \lambda'\boldsymbol{\Lambda} + \boldsymbol{\Lambda}\times\boldsymbol{\Lambda}']\!],\end{aligned} \tag{S-10}$$

and then translated back into the encoding $\hat{C}_{n''}^{k''}(\mathbf{n}'')$ where $\mathbf{n}''$ is normalized and k'', n'' are integers satisfying the same conditions as in Equation (S-2). The integrality of k'' and n'' is guaranteed by the closure of the prevailing finite molecular symmetry group: the composition of two symmetry rotations of a finite molecular system must yield another symmetry rotation of the same system.

S2 Algorithms for character table generation

In this Section, the algorithms for automatic character table computation implemented in QSym^2 are briefly described to highlight their main ideas. The readers are referred to the references therein for more detailed and technical descriptions.

S2.1 The eigenvalue problem for characters

Consider a group $\mathcal{G}$ with k unitary conjugacy classes and hence k irreducible representations. For any conjugacy classes K_r, K_s, and K_t, and for *any* $g_t \in K_t$, define

$$n_{rst} = |\{(g_r, g_s) \in K_r \times K_s : g_r g_s = g_t\}|,$$

which can be shown to be *independent of the choice of* $g_t \in K_t$. The numbers n_{rst} can be gathered into k *class matrices* whose entries are all positive integers:

$$\mathbf{N}_r = [n_{rst}] \in \mathbb{N}^{k^2}, \quad r = 1, \ldots, k.$$

The k class matrices can be determined efficiently from the group's Cayley table using Schneider's method[S4] and are key to the determination of irreducible representation characters, as shall be seen shortly.

Next, let ρ_i be the ith irreducible representation of $\mathcal{G}$ with the corresponding character function χ_i. For the conjugacy class K_j, define

$$\omega_{ij} = \frac{|K_j|\chi_i(K_j)}{\chi_i(K_1)},$$

where $|K_j|$ denotes the size of K_j and K_1 the identity class of $\mathcal{G}$. It can then be shown that

$$\sum_t n_{rst}\omega_{it} = \omega_{ir}\omega_{is}, \tag{S-11a}$$

or, in matrix notations,

$$\mathbf{N}_r \boldsymbol{\omega}_i = \omega_{ir}\boldsymbol{\omega}_i, \tag{S-11b}$$

and

$$\sum_{r=1}^{k} \frac{\omega_{ir}\omega_{jr'}}{|K_r|} = \frac{\delta_{ij}|\mathcal{G}|}{\chi_i(K_1)\chi_j(K_1)}, \tag{S-12}$$

where $K_{r'} = K_r^{-1}$ is the inverse conjugacy class of K_r (*i.e.*, the elements in $K_{r'}$ are all inverses of the elements in K_r). Equation (S-11) indicates that the $\boldsymbol{\omega}_i$'s are all eigenvectors of each $\mathbf{N}_r$, and that the ω_{ir}'s are also eigenvalues of $\mathbf{N}_r$. Therefore, in principle, as long as any one of the k class matrices has been computed, its linearly independent eigenvectors can be found and, with the help of Equation (S-12), characters of all irreducible representations in the group can be determined. This is known as the Burnside algorithm.[S5]

S2.2 Diagonalization over a finite field

The fact that the ω_{ir}'s are eigenvalues of $\mathbf{N}_r$ means that they must be roots of the characteristic polynomials of the class matrices:

$$p_{\mathbf{N}_r}(\lambda) = \det(\lambda\mathbf{I} - \mathbf{N}_r) = 0.$$

But since the entries of the class matrices are all integers, the polynomials $p_{\mathbf{N}_r}(\lambda)$ must all have integer coefficients, which implies that their roots, *i.e.*, the ω_{ir}'s, must be *algebraic integers*. Additionally, it can be shown that the characters χ_i are algebraic integers that can be written as sums of roots of unity. Therefore, algorithms that do not make use of this property and instead only naïvely diagonalize the class matrices over the complex field $\mathbb{C}$ end up doing more work than needed and thus suffer from an unnecessary reduction of efficiency and accuracy.

Recognising these properties and problems, Dixon[S5,S6] proposed a variation to the Burnside algorithm in which all expensive algebras in the character table construction for the group $\mathcal{G}$ are performed in a finite number field of prime order denoted $\mathsf{GF}(p)$, where p is a prime number determined by the orders of the elements in $\mathcal{G}$. $\mathsf{GF}(p)$ can be most simply constructed as the field of integers modulo p, denoted $\mathbb{Z}/p\mathbb{Z}$. The finiteness of $\mathsf{GF}(p)$ allows all arithmetic operations, especially division, to be performed exactly, since their outcomes must reside within the field due to the field's closure properties. In QSym^2, modular arithmetic over $\mathsf{GF}(p)$ is carried out efficiently by making use of the Montgomery form[S7] and eigenvectors of class matrices are determined by Gaussian elimination. Once character values have been calculated exactly in $\mathsf{GF}(p)$, they can be stored, represented, and manipulated symbolically as sums of roots of unity, and are only lifted back to $\mathbb{C}$ when necessary.

S3 Symmetry of electron densities

Table S1: Electronic wavefunction and total density symmetries of the lowest $M_S = -1$ state of H_3^+. Calculations were performed at the UHF and r^{2}SCAN0 levels of theory in the 6-311++(2+,2+)G** basis set. The magnitudes of the applied electric fields and magnetic fields are 0.1 a.u. and $1.0B_0$, respectively. Isosurfaces of total densities are plotted at $|\rho(\mathbf{r})| = 0.050$.

	Field	Symmetry group	Wavefunction symmetry	Density symmetry	Density isosurface
UHF	$\mathbf{0}$	$\mathcal{D}_{3h}$	$A_1' \oplus E'$	$A_1' \oplus E'$	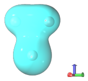
	E_x	$\mathcal{C}_{3v}$	$A_1 \oplus E$	$A_1 \oplus E$	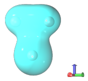
	E_y	$\mathcal{C}_s$	A'	A'	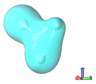
	E_z	$\mathcal{C}_{2v}$	A_1	A_1	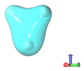
	B_x	$\mathcal{C}_{3h}$	Γ'	A'	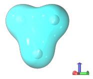
	B_y	$\mathcal{C}_s$	A'	A'	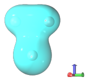
	B_z	$\mathcal{C}_2$	B	A	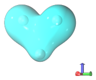
r^{2}SCAN0	$\mathbf{0}$	$\mathcal{D}_{3h}$	–	$A_1' \oplus E'$	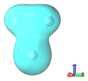
	E_x	$\mathcal{C}_{3v}$	–	$A_1 \oplus E$	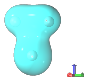
	E_y	$\mathcal{C}_s$	–	A'	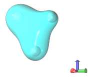
	E_z	$\mathcal{C}_{2v}$	–	A_1	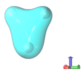
	B_x	$\mathcal{C}_{3h}$	–	A'	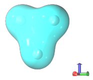
	B_y	$\mathcal{C}_s$	–	A'	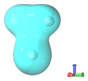
	B_z	$\mathcal{C}_2$	–	A	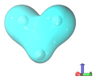

References

(S1) Ceulemans, A. J. *Group Theory Applied to Chemistry*; Springer Science & Business Media, 2013.

(S2) Altmann, S. L. *Rotations, Quaternions, and Double Groups*; Dover Publications, Inc.: New York, 2005.

(S3) Kolmogorov, A. N.; Fomin, S. V. *Introductory Real Analysis*; Dover Publications, Inc.: New York, 1970.

(S4) Schneider, G. J. Dixon's Character Table Algorithm Revisited. *J. Symb. Comput.* **1990**, *9*, 601–606.

(S5) Grove, L. C. *Groups and Characters*; John Wiley & Sons, Inc.: New York, United States, 1997.

(S6) Dixon, J. D. High Speed Computation of Group Characters. *Numer. Math.* **1967**, *10*, 446–450.

(S7) Montgomery, P. L. Modular Multiplication without Trial Division. *Math. Comp.* **1985**, *44*, 519–521.
